# Prevalence and Antimicrobial Resistance of *Enterobacteriaceae* in Wild Birds Across Europe: A Systematic Review

**DOI:** 10.3390/antibiotics14090905

**Published:** 2025-09-08

**Authors:** Daiana-Ionela Cocoș, Eugenia Dumitrescu, Florin Muselin, Diana Brezovan, János Degi, Oana-Maria Boldura, Romeo T. Cristina

**Affiliations:** 1 Department of Pharmacy and Pharmacology, University of Life Science “King Michael I”, 300645 Timisoara, Romania; eugeniadumitrescu@usvt.ro; 2 Department of Toxicology, University of Life Science “King Michael I”, 300645 Timisoara, Romania; florinmuselin@usvt.ro; 3 Department of Histology and Cell Biology, University of Life Science “King Michael I”, 300645 Timisoara, Romania; dianabrezovan@usvt.ro; 4 Department of Infectious Diseases and Preventive Medicine, University of Life Science “King Michael I”, 300645 Timisoara, Romania; janosdegi@usvt.ro; 5 Department of Biochemistry, University of Life Science “King Michael I”, 300645 Timisoara, Romania; oanaboldura@usvt.ro

**Keywords:** wild birds, *Enterobacteriaceae*, antimicrobial resistance, One Health, Europe

## Abstract

Wild birds are increasingly recognized as reservoirs and vectors of antimicrobial-resistant (AMR) *Enterobacteriaceae*, but comprehensive assessments across Europe remain limited. AMR represents a growing threat to global health under the One Health framework. **Background/Objectives:** This review aimed to evaluate the occurrence, diversity, and resistance patterns of *Enterobacteriaceae* in wild birds across Europe (1969–2025), and to identify ecological and methodological trends. **Methods:** Following Preferred Reporting Items for Systematic Reviews and Meta-Analyses (PRISMA) 2020 guidelines, we searched PubMed and Web of Science until July 2025. Inclusion criteria targeted studies reporting *Enterobacteriaceae* isolation and/or AMR in free-living European birds. Data were synthesized thematically by bacterial species, avian order, resistance profile, and country. Risk of bias was assessed based on sampling, reporting, and diagnostic clarity. **Results:** Eighty studies met the inclusion criteria, covering over 18,000 wild birds from 25 countries. *Escherichia coli* and *Salmonella enterica* were most reported, often exhibiting resistance to β-lactams, fluoroquinolones, and tetracyclines. AMR was detected in birds from both urban and natural areas. Study designs varied widely, with inconsistent methods for bacterial identification and susceptibility testing. **Conclusions:** Wild birds in Europe carry resistant *Enterobacteriaceae*, including strains with clinically relevant resistance profiles. These findings support their inclusion in One Health AMR surveillance and highlight the need for harmonized protocols, expanded molecular tools, and ecological integration.

## 1. Introduction

The increasing spread of antimicrobial resistance (AMR) is one of the most critical global health challenges of the 21st century, posing a pressing concern across human, veterinary, and environmental health sectors [1,2,3,4].

Among the most clinically significant AMR organisms are members of the *Enterobacteriaceae* family, including *Escherichia coli*, *Salmonella enterica*, *Klebsiella pneumoniae*, *Proteus* spp., and *Enterobacter* spp., which are frequently implicated in zoonotic infections and are increasingly isolated from non-traditional reservoirs, including wild fauna [5,6,7,8,9].

Although AMR surveillance systems in Europe have traditionally focused on clinical and livestock settings [3,10], the role of wildlife, particularly wild birds, has received growing attention in recent decades [11,12]. Wild birds exhibit significant movement across broad geographical areas and often occupy habitats influenced by human activities, including urban parks, agricultural landscapes, and landfills. These ecological characteristics make them ideal sentinels for environmental AMR and potential reservoirs or vectors of resistance bacteria [13,14,15].

Numerous studies have documented the presence of extended-spectrum beta-lactamase (ESBL)-producing *Escherichia coli*, plasmid-mediated quinolone resistance (PMQR), and multidrug-resistant phenotypes in a wide range of bird species, including gulls, pigeons, raptors, and passerines across Europe [14,16,17,18,19,20].

These findings underscore critical concerns for environmental AMR surveillance and highlight the One Health relevance of interactions at the wildlife–livestock–human interface [21,22,23,24,25].

Despite substantial scientific efforts, the current research landscape remains fragmented, reflecting methodological inconsistencies, imbalances in sampling across regions and avian taxonomy, and a scarcity of longitudinal investigations. Furthermore, the ecological, taxonomic, and behavioral heterogeneity of wild birds is frequently neglected in meta-analyses, thereby limiting the robustness of species-specific risk assessments. Therefore, the purpose of this paper is to comprehensively synthesize the available evidence on the identification of *Enterobacteriaceae* and the characterization of AMR in wild birds across Europe.

## 2. Results

The results of this systematic review are structured according to the taxonomic order of avian hosts from which *Enterobacteriaceae* were isolated. Each subsection presents the relevant data based on the country of sampling, the investigation period, and the number of positive birds, often accompanied by the percentage relative to the total number of birds examined, where available. This classification allows a clearer comparative analysis between different avian orders and geographical or temporal patterns of bacterial detection.

### 2.1. Accipitriformes

A total of 25 studies, published from 1969 to 2024, reported data on *Enterobacteriaceae* isolation in birds of the order *Accipitriformes*, conducted across 10 countries.

Refsum et al. [17] detected in Norway three positive birds in a study conducted over three decades (1969–2000), although the total number of individuals examined was not disclosed. In contrast, Skov et al. [26] did not find any bacteria among two examined individuals in Denmark during 2001–2002.

The largest proportion of studies (48%, 12/25) were conducted in Spain, demonstrating significant variability in prevalence across time and region. Early work by Reche et al. [27] identified a 5.7% prevalence rate (10/176), whereas Millán et al. [21] reported a higher prevalence of 13.34% (4/30). More recent studies revealed increasing rates: Marín et al. [28] found a 52.6% prevalence (51/97), Alcalá et al. [29] recorded 10.2% (5/49), Jurado-Tarifa et al. [30] recorded 4.82% (4/83), and Molina-López et al. [31] observed 100% (2/2) but in a very small sample. In 2016, Blanco et al. [32] and Marin et al. [33] reported comparable prevalence rates of 34.2% (39/114) and 21.1% (22/104), respectively. Martin-Maldonado et al. [34] and Oteo et al. [35] both confirmed high infection rates in 2015–2016, reporting 45.2% (28/62) and an unspecified sample size with 39 positives. Tardón et al. [36] found a 90% prevalence (9/10) from buffers on the bone surface of fractures, and lastly, the most recent study by Guitart-Matas et al. [12] found a 39.9% prevalence (87/218), consolidating Spain’s status as a hotspot for *Enterobacteriaceae* in this bird order.

In Germany, four studies confirmed consistently high rates. Guenther et al. [14] reported a 95.38% prevalence (62/65) between 2006 and 2008, while Guenther et al. [37] reported a prevalence of 38% (65/171). Fischer et al. [38] observed 100% prevalence in a small sample, and Schaufler et al. [39] detected seven positives, though without specifying the total number tested.

Studies from Italy revealed variable prevalence [6,40,41,42]. Botti et al. [42] did not specify the total number of examined birds, while Gargiulo et al. [41] observed 10.86% prevalence (5/46), offering the only quantified insight from this country.

In the United Kingdom, Pennycott et al. [43] found 9.09% prevalence from 11 birds. Veldman et al. [44] in the Netherlands reported 0% prevalence in 18 tested individuals (2010–2011). Similarly, Krawiec et al. [45] in Poland reported a 16.67% prevalence.

Konicek et al. [46] conducted studies in both Austria and the Czech Republic, examining 31 and 47 birds, respectively, but without providing information on the cases of this order.

Handrová and Kmet [7] reported 100% prevalence in Slovakia, where all 19 tested birds were positive—one of the highest rates documented. Lastly, Zurfluh et al. [47] in Switzerland identified three positives, but the total number of birds tested remains unknown.

**Figure 1:**
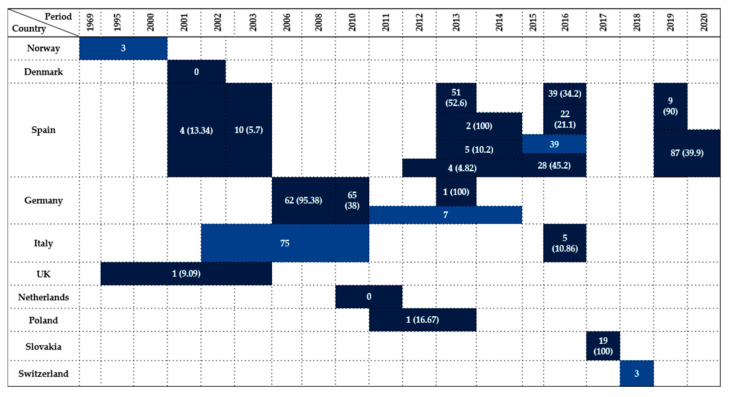
Visual representation of *Enterobacteriaceae* prevalence in *Accipitriformes* by country and year. Darker colored cells indicate positive findings with prevalence values in parentheses, while lighter colored cells indicate positive findings without prevalence data (number of isolates only).

Taken together, the highest prevalence rates were recorded in Central Europe (Germany, Slovakia), underscoring possible ecological or anthropogenic influences on exposure. Most isolates in this order were obtained from cloacal swabs and fecal samples, with occasional use of postmortem tissues, pellets, or bone surface buffers. These sampling approaches were most frequently applied in Central and Southern Europe, whereas northern regions contributed only sporadic detections. These data are synthesized in Figure 1, which illustrates prevalence by country and year.

### 2.2. Falconiformes

Twelve studies reported data on *Enterobacteriaceae* detection in wild birds of the order *Falconiformes* across five European countries between 2001 and 2019. Prevalence rates varied widely (0–50%), depending on year, sampling method, and host species.

Spain was the most represented country, contributing seven studies (58% of total), where the most studied bird was the Common kestrel (*Falco tinnununculus*). The earliest and largest survey was conducted by Reche et al. [27], who reported a 4.34% prevalence (13/300) over a general 3-year period. Subsequent studies displayed varied findings: Millán et al. [21] recorded a 20% prevalence (1/5) in 2001–2002, and Molina-López et al. [31] found a 12.5% (2/16) prevalence in 2013–2014. In contrast, Alcalá et al. [29] reported no prevalence from eight samples during the same period of time. Between 2012 and 2014, Jurado-Tarifa et al. [30] observed a 5.88% prevalence rate (5/85), while Oteo et al. [35] detected six positives in a 2015–2016 survey without reporting the sample size. The most recent study by Tardón et al. [36] revealed a high prevalence of 33.33% (1/3) in 2019, but the type of sample was taken from surface of fractures.

From Italy, several *Falco* species were investigated and available in four studies [6,40,41,42], but just two of them reported relevant results. Botti et al. [42] detected two positive birds during 2002–2010, though the total sample size of this bird’s order remains unspecified. Gargiulo et al. [41], however, provided the most conclusive data, identifying a prevalence of 17% (10/59) in 2016.

In Germany, Guenther et al. [14] observed a 50% prevalence rate (6/12) in birds sampled between 2006 and 2008.

By contrast, Veldman et al. [44] in the Netherlands found no *Enterobacteriaceae* among one bird sampled during 2010–2011.

In Austria and the Czech Republic, Konicek et al. [46] examined 68 and 15 birds, respectively, in 2013–2014, detecting *Enterobacteriaceae* but not reporting prevalence.

Finally, Zurfluh et al. [47] from Switzerland detected one positive Eurasian hobby (*Falco subbuteo*) in 2018.

Overall, results indicate heterogeneous *Enterobacteriaceae* detection in *Falconiformes* across Europe, influenced by regional, ecological, and methodological variables. Sampling relied mainly on cloacal swabs and fecal samples, with postmortem tissues, pellets, and bone surface buffers used sporadically, particularly in Central and Southern Europe.

**Figure 2:**
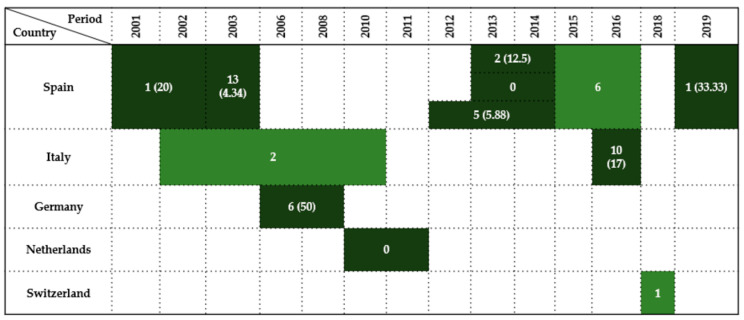
Visual representation of *Enterobacteriaceae* prevalence in *Falconiformes* by country and year. Darker colored cells indicate positive findings with prevalence values in parentheses, while lighter colored cells indicate positive findings without prevalence data (number of isolates only).

A summary of findings by country and investigation period is provided in Figure 2.

### 2.3. Strigiformes

Thirteen studies examined the presence of *Enterobacteriaceae* in wild birds belonging to the order *Strigiformes*, covering five European countries between 1969 and 2019. Prevalence rates varied widely, from 4.69% to 100%, reflecting differences in bird species, environmental exposure, and sampling design.

In Norway, Kapperud & Rosef [48] reported a prevalence of 16.7% (2/12) in *Aegolius funereus* during 1980–1981, based on cloacal swabs. A broader retrospective study by Refsum et al. [17], covering the years 1969–2000, detected a single positive owl.

The majority of data originated from Spain, which contributed 54% of the total studies (7/13). Reche et al. [27] performed a comprehensive 3-year survey of 119 owls across several species, reporting a 10.1% prevalence. In a smaller study, Millán et al. [21] recorded 20% (1/5) during 2001–2002. Between 2012 and 2014, Jurado-Tarifa et al. [30] analyzed 192 birds, which resulted in a 4.69% prevalence, while Molina-López et al. [31] found a prevalence of 12% (3/25) during 2013–2014. In the same period, Alcalá et al. [29] identified only a 5.9% (1/17) prevalence. Later studies revealed even higher rates: Oteo et al. [35] detected eight positive individuals in 2015–2016, and Tardón et al. [36] observed a 80% (4/5) prevalence in 2019, representing one of the highest prevalence rates across all countries.

Pennycott et al. [43] reported a prevalence of 50% (2/4) in tawny owls sampled between 1995 and 2003, based on postmortem analysis in the UK.

Germany recorded the highest prevalence in this bird order: Guenther et al. [14] observed *Escherichia coli* in all 10 owls tested (100%) between 2006 and 2008 using cloacal swabs and postmortem sampling.

Three studies were conducted in Italy, representing 23% (3/13) of the total dataset. Botti et al. [42] detected six positive owls between 2002 and 2010, although no total number of examined individuals was given; likewise, Dipineto et al. [40] examined 13 owls but did not specified the positive number, if they had one. Gargiulo et al. [41] provided the most complete data, identifying a 11.62% prevalence (5/43) in the 2015–2016 period.

**Figure 3:**
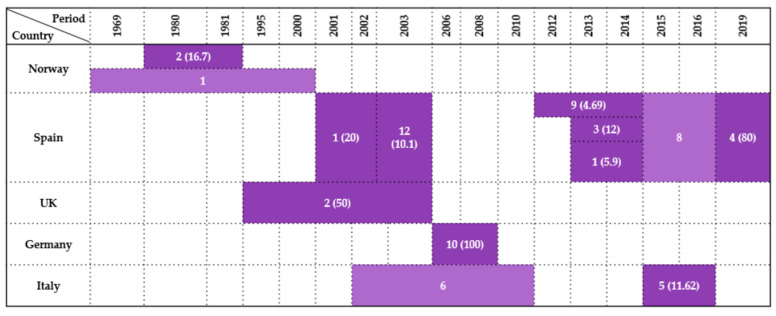
Visual representation of *Enterobacteriaceae* prevalence in *Strigiformes* by country and year. Darker colored cells indicate positive findings with prevalence values in parentheses, while lighter colored cells indicate positive findings without prevalence data (number of isolates only).

These findings underscore the considerable variability in *Enterobacteriaceae* prevalence among *Strigiformes* across Europe, shaped by ecological conditions, species susceptibility, and diagnostic methods. Cloacal swabs and fecal samples predominated, particularly in Central and Southern Europe, while other occasional sample types were used only sporadically. A visual synthesis of the results is presented in Figure 3.

### 2.4. Columbiformes

Twenty-four studies from 14 countries assessed the presence of *Enterobacteriaceae* in wild *Columbiformes* between 1969 and 2019. Prevalence ranged from 0% to 100%, with substantial variability attributed to ecological, geographical, and methodological factors.

In Norway, three studies were conducted. Kapperud & Rosef [48] did not report any *Enterobacteriaceae* among 71 birds tested during 1980–1981. In contrast, Refsum et al. [17] reported a low prevalence of 4.17% (3/72) between 1969 and 2000. Later, Lillehaug et al. [49] tested 200 birds in 2003 and again did not find any results. These contrasting results suggest either temporal fluctuations or localized absence of infection.

The Czech Republic contributed two investigations with starkly different outcomes. Cízek et al. [50] recorded only a 0.46% prevalence (2/432) between 1984 and 1991, while Konicek et al. [46] later reported an extremely high prevalence of 92.1% (281/305) in 2013–2014, indicating a potential shift in pathogen circulation or diagnostic sensitivity over time.

Italy accounted for 12.5% of all studies (3/24). Morabito et al. [51] identified a 10.7% prevalence (70/649) in 1997–1998. Later, Botti et al. [42] detected four positive cases during 2002–2010, without providing a total sample size, and Rubini et al. [52] reported zero cases from five birds tested between 2010 and 2013.

In Finland, Kobayashi et al. [16] found a 7% prevalence (2/29) in 1998, while Skov et al. [26] reported 0 cases in 11 pigeons tested in Denmark during 2001–2002.

Pennycott et al. [43] in Scotland recorded a prevalence of 5.75% (5/87) between 1995 and 2003, while in the broader United Kingdom, Hughes et al. [53,54] detected two positives in a 2006 survey.

In Germany, Guenther et al. [14] reported 100% prevalence (20/20) between 2006 and 2008, the highest among all studies, while Schaufler et al. [39] later detected just one positive bird in the period of 2011–2014.

From Spain, three studies were published. Andrés et al. [55] detected two positives between 2009–2011, and the same Alcalá et al. [29] found no cases in five pigeons sampled during 2013–2014, while Martin-Maldonado et al. [56] reported a 7% prevalence (7/100) in 2018–2019.

In the Netherlands, Veldman et al. [44] reported a 14.3% prevalence rate (1/7) between 2010 and 2011. In Switzerland, Zurfluh et al. [57] found a low prevalence of 1% (3/298) in 2012, whereas a later report from 2018 [47] detected four additional positive birds.

Konicek et al. [46] reported a prevalence of 63.7% (165/259) in Austria during 2013–2014. In Poland, Krawiec et al. [45,58] identified a 16.67% prevalence (1/6) between 2011 and 2013.

Outside continental Europe, Tessier et al. [59] found no *Enterobacteriaceae* among 30 *Columbiformes* sampled on Réunion Island (2011–2013). In France, Ngaiganam et al. [60] recorded a 4.22% prevalence rate (3/71) in 2016.

**Figure 4:**
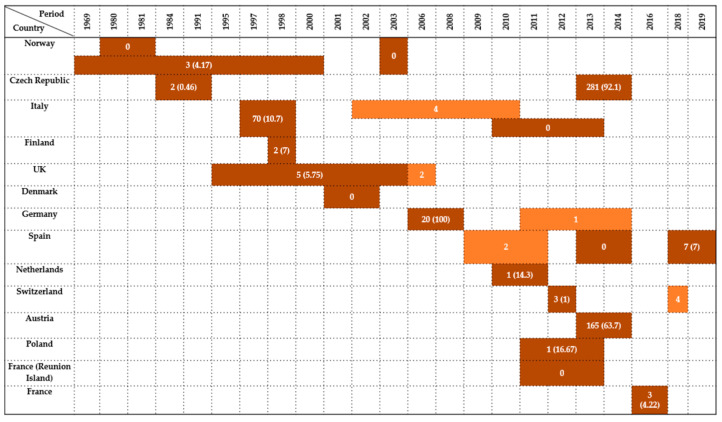
Visual representation of *Enterobacteriaceae* prevalence in *Columbiformes* by country and year. Darker colored cells indicate positive findings with prevalence values in parentheses, while lighter colored cells indicate positive findings without prevalence data (number of isolates only).

Taken together, the highest prevalence rates were observed in Central Europe (Germany, the Czech Republic, Austria), where values ranged from 63.7% to 100% in *Columbiformes* species such as the Eurasian collared dove (*Streptopelia decaocto*), rock pigeon (*Columba livia*), common wood pigeon (*Columba palumbus*); in contrast, studies from Northern Europe and peripheral regions reported zero detection. These differences highlight the influence of local ecology, species susceptibility, and testing methodologies. Most isolates originated from cloacal swabs and fecal samples, whereas postmortem material was only occasionally analyzed. A summary of the findings is presented in Figure 4.

### 2.5. Passeriformes (Corvidae)

Twenty-four records from 13 countries reported data on *Enterobacteriaceae* isolation in wild birds from the *Corvidae* family between 1969 and 2023. Prevalence rates ranged widely from 0% to 81.81%, reflecting considerable variation in study design, geographic context, and sample sizes.

In Norway, three studies captured different time points and infection dynamics. Refsum et al. [17] reported a low prevalence of 2.17% (2/92) in birds sampled over a broad period (1969–2000). By contrast, Kapperud & Olsvik [61] documented a high prevalence of 50% (2/4) in 1979, while Kapperud & Rosef [48] observed no prevalence among 50 individuals during 1980–1981.

Pennycott et al. [43] identified a 3.63% prevalence (2/55) collected between 1995 and 2003 in the UK.

Three studies were conducted in Spain. Millán et al. [21] reported no cases from seven birds sampled during 2001–2002. A decade later, Janecko et al. [62] found a low prevalence of 1.34% (2/150) in 2011, while Oteo et al. [35] detected a single positive case from 2015–2016.

Skov et al. [26] in Denmark observed 0% prevalence in 2001–2002.

In Italy, four studies provided data. Botti et al. [42] detected two positive individuals from 2002–2010. In 2011, Janecko et al. [62] found 0% prevalence among 150 birds. Rubini et al. [52] documented a prevalence of 1.08% (9/831) between 2010 and 2013, while Giacopello et al. [6] investigated four individuals in 2013 but did not report prevalence.

Germany presented notable contrasts across three time points. Guenther et al. [14] found a high prevalence of 81.81% (18/22) between 2006 and 2008. In 2011, a 1% prevalence was reported (1/100) [62], while Schaufler et al. [39] detected three more positive individuals between 2011 and 2014

In the Netherlands, Veldman et al. [44] tested a single bird (2010–2011) and reported no prevalence.

In the Czech Republic, a prevalence of 0.76% (4/525) was recorded between 2010 and 2013, while in France, a 9.68% (3/31) prevalence was recorded in 2011 [62].

Janecko et al. [62] also conducted studies in Serbia, Poland, Switzerland, and Slovakia. In Serbia, a 0% prevalence was recorded among 150 birds; while Slovakia, a 3.15% prevalence (9/286) was recorded in 2013. In Switzerland, a 0% prevalence was reported in 49 birds sampled in 2011 [62], whereas Zurfluh et al. [47] later detected 2 positives in 2018.

In Poland, a total of three studies were available: The first one reported a 0.67% prevalence (2/298) in 2011 [62], Krawiec et al. [45] found a 8.33% prevalence (1/12) from 2011 to 2013, and Łopucki et al. [63] documented a markedly higher prevalence of 52% (31/60) during 2022–2023.

Finally, Konicek et al. [46] examined 130 Corvidae species in Austria between 2013 and 2014, though no positive results were detected.

**Figure 5:**
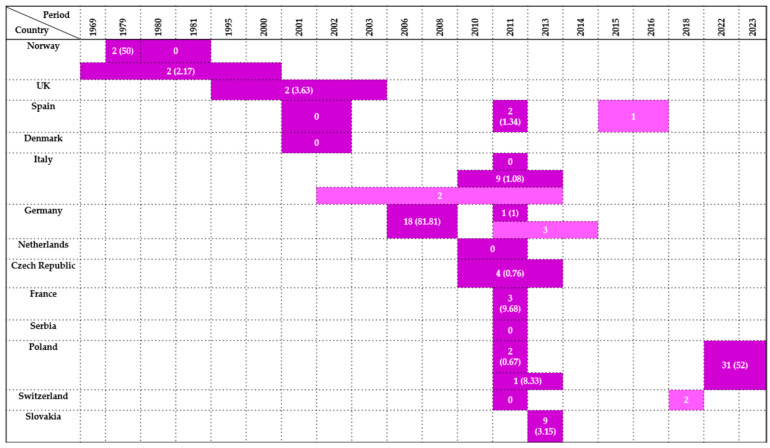
Visual representation of *Enterobacteriaceae* prevalence in *Corvidae* by country and year. Darker colored cells indicate positive findings with prevalence values in parentheses, while lighter colored cells indicate positive findings without prevalence data (number of isolates only).

Overall, the highest *Enterobacteriaceae* prevalence rates in *Corvidae* were observed in Central Europe, particularly in Germany (81.8%) and Poland (52%), while Northern and Southern European countries generally reported either no or minimal detection. These discrepancies reflect differences in diagnostic sensitivity, sampling intensity, regional ecology, and potential antimicrobial resistance patterns. Most isolates were obtained from fecal and cloacal swabs, whereas postmortem was only occasionally used. The results are synthesized visually in Figure 5.

### 2.6. Charadriiformes (Laridae)

A total of 28 studies focused on *Enterobacteriaceae* occurrence in wild birds of the family *Laridae*, involving 14 European countries over a period ranging from 1969 to 2020. Considerable variation was observed in the number of positive birds and prevalence rates across regions and years.

In Norway, four studies provided insight into temporal trends. Kapperud & Rosef [48] reported a 2.8% prevalence (6/216) during 1980–1981. Earlier, Refsum et al. [17] detected 15 positive birds from 1969–2000. Nesse et al. [64] recorded a prevalence of 7.34% (31/422) during 2000–2001, and a more recent investigation by Literak et al. [65] found a 13% prevalence (2/15).

From the United Kingdom, Pennycott et al. [43] recorded a 7.21% prevalence (7/97) in Scotland between 1995 and 2003. Hughes et al. [53,54] detected four positive gulls in 2005, and another study did not specify either the number of isolates or the total examined birds [66].

In Switzerland, Zurfluh et al. [47] detected one positive gull in 2018.

The Czech Republic contributed three major datasets. Cízek et al. [50] found a 16.24% prevalence (151/930) between 1984 and 1991. Hubálek et al. [67] recorded a higher prevalence of 24.7% (38/154) in 1992–1993. More recently, Nesporova et al. [68] reported a prevalence of 67.5% (79/117) sampled during 2018–2019.

In Sweden, studies revealed increasing prevalence over time. Palmgren et al. [69] observed a 4% prevalence (2/50) in 1997, and Wahlström et al. [70] found a prevalence of 3.6% (4/111) in 1998–1999. A dramatic increase was noted by Bonnedahl et al. [71] in 2008, with 83% prevalence out of 100. Later, Atterby et al. [23] recorded a prevalence of 17% (29/170) in 2013.

In Finland, Kobayashi et al. [16] identified a 40% prevalence (34/86).

Skov et al. [26] in Denmark examined two gulls in 2001–2002, with 0% prevalence reported.

In France, Bonnedahl et al. [13] recorded one of the highest prevalence rates: 85% (153/180) in 2009, while Ngaiganam et al. [60] reported 24.32% (9/37) in 2016.

Two German studies provided contrasting findings. Guenther et al. [14] identified a 50% prevalence (1/2) in 2006–2008, while Schaufler et al. [39] detected one additional positive bird between 2011 and 2014.

In Poland, Literak et al. [72] recorded a prevalence of 66.7% (18/27).

In Italy, Botti et al. [42] detected a single positive gull from their investigation between 2002 and 2010.

In the Netherlands, Veldman et al. [44] found a prevalence of 18.83% (29/154) during 2010–2011.

Ireland showed consistently high prevalence, with Carroll et al. [73] reporting a 77.8% prevalence (70/90) in 2013.

Four studies were available from Spain. Alcalá et al. [29] identified a 100% prevalence out of one gull sampled in 2013–2014. Martin-Maldonado et al. [56] reported a prevalence of 20% (5/25) in 2018–2019. Manzanares-Pedrosa et al. [74] aggregated data from 2013, 2009–2010, and 2018, reporting a 10.5% prevalence (45/429). Additionally, Oteo et al. [35] detected 15 positive individuals during 2015–2016.

**Figure 6:**
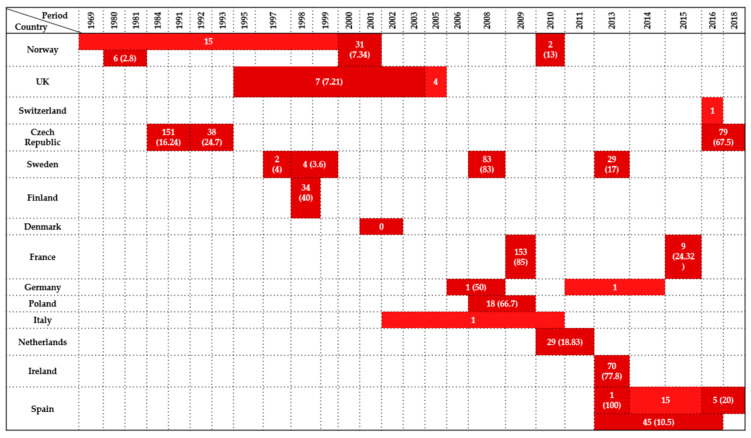
Visual representation of *Enterobacteriaceae* prevalence in *Laridae* by country and year. Darker colored cells indicate positive findings with prevalence values in parentheses, while lighter colored cells indicate positive findings without prevalence data (number of isolates only).

*Enterobacteriaceae* prevalence in Laridae ranged from 0% to 100%, with particularly high rates recorded in Western and Central Europe, including France, Ireland, Sweden, Czech Republic, and Poland. These findings likely reflect a combination of environmental exposure, water contamination, migratory routes, and methodological heterogeneity. Most isolates originated from cloacal swabs and fecal samples, while postmortem and pooled samples were only occasionally analyzed. A summary of the data is presented in Figure 6.

### 2.7. Aquatic Birds

#### 2.7.1. Anseriformes

A total of 24 studies conducted between 1969 and 2024 reported the presence of *Enterobacteriaceae* in wild birds of the order *Anseriformes* across 11 European countries. Prevalence rates ranged from 0% to 100%, reflecting significant differences in methodology, species, and environmental exposure.

In the United Kingdom, two studies examined birds over distinct timeframes. Mitchell & Ridgwell [75] recorded a 4.2% prevalence (20/477) between 1969 and 1970. Decades later, Pennycott et al. [43] found 0% prevalence in 23 individuals sampled during 1995–2003.

Two studies were also available from Norway. Refsum et al. [17] detected four positives in a long-term surveillance program from 1969 to 2000, while Kapperud & Rosef [48] found no *Enterobacteriaceae* among one bird sampled during 1980–1981.

In Sweden, Wahlström et al. [70] observed 0% prevalence among 105 birds in 1998–1999. In contrast, Hessman et al. [76] identified a high prevalence of 47% (386/813), indicating a significant increase in detection over time.

From Spain, 0% prevalence was reported in 278 individuals examined between 2008 and 2014 [29,77].

Five studies originated from Poland, offering diverse results. Literak et al. [72] reported a 75.6% prevalence (65/86) sampled during 2008–2009. Two independent studies from 2011–2013 showed contrasting values: Krawiec et al. [45] recorded a lower prevalence of 5.2% (8/154), while Kuczkowski et al. [78] found 100% prevalence (75/75). Additionally, Krawiec et al. [58] detected five positive birds, and Wodz et al. [79] identified a single case out of one tested (100%) in the most recent dataset.

In Belgium, Garmyn et al. [80] documented only a 0.5% prevalence (2/396) in 2011, one of the lowest rates recorded.

Two studies were conducted in Italy with samples taken from intestinal content and postmortem. Iannibelli et al. [81] found a single case in 1991 (100%), whereas Rubini et al. [52] reported no cases among two birds examined between 2010 and 2013.

In the Netherlands, Veldman et al. [44] observed a prevalence of 18.26% (21/115) during 2010–2011. Later, Kuczkowski et al. [78] reported a 100% rate in a sample of 94 birds during 2011–2013.

Data from Germany revealed high bacterial presence. Rödiger et al. [82] detected 400 positive birds between 2007 and 2011, and Schaufler et al. [39] detected an additional 11 positive cases in the period 2011–2014.

In Austria, Konicek et al. [46] found an 80% prevalence (40/50) during 2013–2014.

From the Czech Republic, two studies offered long-term insight. Hubálek et al. [67] recorded a prevalence of 4.76% (1/21) between 1992 and 1993. A more recent investigation by Konicek et al. [46] reported a 98% prevalence (50/51) in 2013–2014.

Martin et al. [83] provided data from Ireland collected between 2013 and 2021, though without details on the number of positive birds or total examined individuals. Finally, Eckenko et al. [84] investigated 111 species of wild ducks and geese and found a 17% prevalence (19/111).

**Figure 7:**
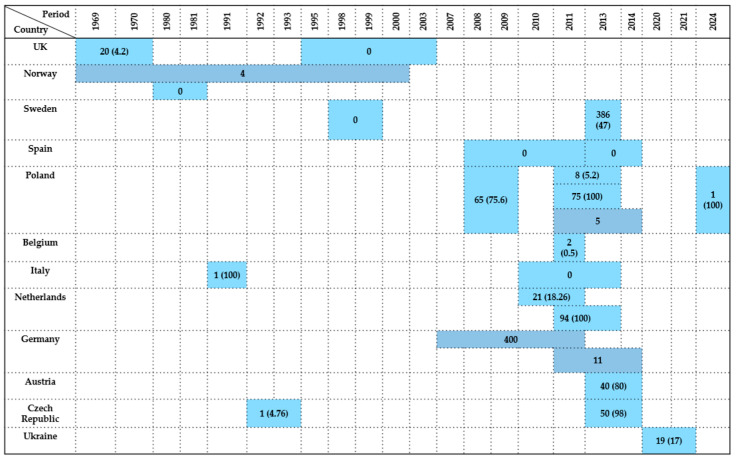
Visual representation of *Enterobacteriaceae* prevalence in *Anseriformes* by country and year. Darker colored cells indicate positive findings with prevalence values in parentheses, while lighter colored cells indicate positive findings without prevalence data (number of isolates only).

In summary, the prevalence of *Enterobacteriaceae* in *Anseriformes* ranged from 0% in several studies from Western and Northern Europe (Spain, Italy, UK, Norway, Sweden) to 100% in select datasets from Central Europe (Poland) and the Netherlands. These differences highlight potential disparities in sampling strategies, laboratory methods, bird species, and habitat-related exposures. Most isolates were obtained from cloacal swabs and fecal samples, while postmortem tissues and intestinal content were analyzed only occasionally. A visual overview is provided in Figure 7.

#### 2.7.2. Pelecaniformes

A total of eight studies conducted between 2001 and 2019 investigated the presence of *Enterobacteriaceae* in wild birds of the order *Pelecaniformes*, with data available from Spain, Italy, the Netherlands, and Germany. Overall, positivity was sporadic and generally low, with limited sample sizes and some missing prevalence data.

In Spain, three investigations covering nearly two decades. Millán et al. [21] and Alcalá et al. [29] both reported 0% prevalence, testing six and two birds during the periods 2001–2002 and 2013–2014, respectively. In contrast, Tardón et al. [36] detected seven positive individuals from a 2019 study, though only two birds were examined because the samples were taken from the bone surface of fractures.

Three datasets were also available from Italy. Botti et al. [42] did not detect positive birds between 2002 and 2010, and Giacopello et al. [6] similarly found no cases in a 2013 study involving three birds. In contrast, Mancini et al. [85] identified a prevalence of 7.7% (1/13) in 2012.

In the Netherlands, Veldman et al. [44] observed a moderate prevalence of 18.18% (2/11) during 2010–2011.

Finally, in Germany, Schaufler et al. [39] detected one positive bird during the 2011–2014 investigation period, although the number of individuals tested was not disclosed.

**Figure 8:**
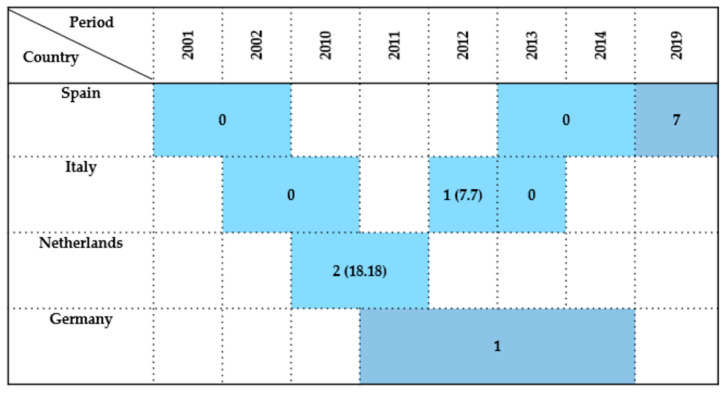
Visual representation of *Enterobacteriaceae* prevalence in *Pelecaniformes* by country and year. Darker colored cells indicate positive findings with prevalence values in parentheses, while lighter colored cells indicate positive findings without prevalence data (number of isolates only).

In summary, *Enterobacteriaceae* detection in *Pelecaniformes* was occasional, with only a few positive cases reported in studies from Southern and Central Europe. Most isolates were obtained from cloacal swabs and, less frequently, from fecal samples, while postmortem tissues and bone surface buffers were only sporadically analyzed. A visual representation of the findings is provided in Figure 8.

#### 2.7.3. Ciconiiformes

A total of 13 studies conducted between 2001 and 2019 investigated the occurrence of *Enterobacteriaceae* in wild birds of the order *Ciconiiformes*, with data reported from Spain, Italy, the Netherlands, Switzerland, Austria, the Czech Republic, and Poland. The prevalence of bacterial detection varied substantially across countries and time periods.

Spain provided the most extensive dataset on the white stork (*Ciconia ciconia*). Millán et al. [21] found no cases among three birds sampled between 2001 and 2002. In a later study, Camacho et al. [86] reported a high prevalence of 95% (114/120) in 2013. Concurrently, Alcalá et al. [29] reported a 33.33% prevalence in nine birds sampled during 2013–2014. More recent studies by Martín-Maldonado et al. [56] and Tardón et al. [36] recorded a 22% prevalence in 100 birds (2018–2019) and eight bacteria isolates in four birds (2019), respectively.

From Italy, Giacopello et al. [6] documented a single case in 2013 (100%), although this value represents a sample size of just one.

In the Netherlands, Veldman et al. [44] found 0% prevalence among seven birds sampled in 2010–2011, while Zurfluh et al. [57] in Switzerland reported a low prevalence of 6.7% (2/30) during 2011–2012.

In Austria, Konicek et al. [46] observed a prevalence of 41.67% (5/12) during 2013–2014. The same study in the Czech Republic [46] reported a notably high prevalence of 73.91% (17/23).

Poland contributed with three datasets. Krawiec et al. [45] reported a 10.4% prevalence (8/77) between 2011 and 2013. Kuczkowski et al. [78] identified a 100% prevalence (21/21) from the same period, while Krawiec et al. [58] detected a single positive bird from 2011–2014.

**Figure 9:**
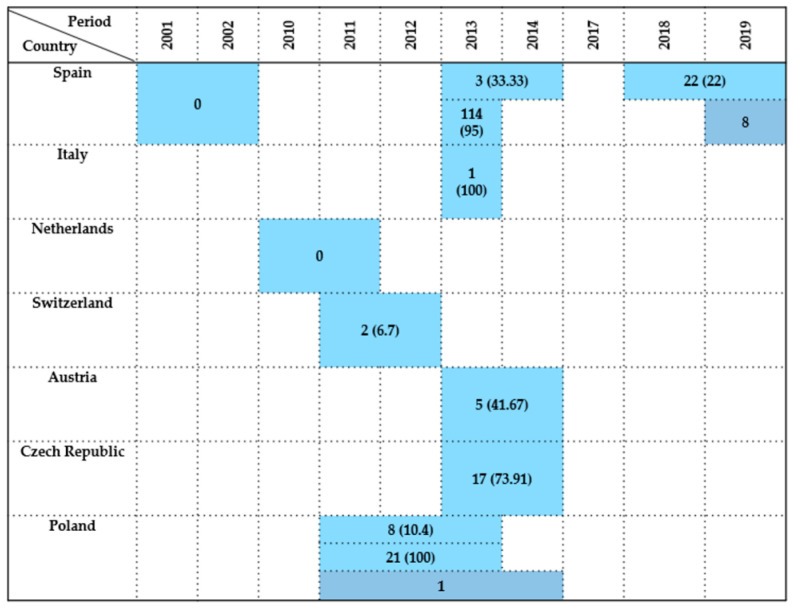
Visual representation of *Enterobacteriaceae* prevalence in *Ciconiiformes* by country and year. Darker colored cells indicate positive findings with prevalence values in parentheses, while lighter colored cells indicate positive findings without prevalence data (number of isolates only).

Overall, prevalence rates in *Ciconiiformes* ranged from 0% to 100%. Higher prevalence was particularly notable in Southern and Central Europe. However, other studies reported no or minimal detection, likely influenced by differences in sample size, species composition, ecological context, and methodological approaches. Most isolates were obtained from cloacal swabs, while postmortem material, fecal samples, and bone surface buffers were used only occasionally. A graphical summary of the findings is provided in Figure 9.

#### 2.7.4. Gruiformes

A total of eight studies examined *Enterobacteriaceae* occurrence in wild birds of the order *Gruiformes*, with data spanning from 1991 to 2014 and covering six European countries. Although the number of investigations was limited, reported prevalence rates varied notably across regions and time periods.

In Italy, Iannibelli et al. [81] identified a prevalence of 10% (1/10).

In the Czech Republic, Hubálek et al. [67] reported a prevalence of 33.33% among three individuals sampled between 1992 and 1993.

In Spain, three studies found no evidence of *Enterobacteriaceae*. Millán et al. [21] tested one individual in 2001–2002, Antilles et al. [77] analyzed a group of 41 birds between 2008 and 2011, and Alcalá et al. [29] likewise reported zero prevalence, but from a single bird examined in 2013–2014.

Consistent with these findings, negative results were also obtained in the Netherlands and Poland. Veldman et al. [44] tested 15 birds in the Netherlands during 2010–2011, and Krawiec et al. [45] examined seven individuals in Poland between 2011 and 2013—both studies reported 0% prevalence.

In contrast, Austria showed a different outcome, with Konicek et al. [46] documenting a 100% prevalence (3/3) during 2013–2014.

**Figure 10:**
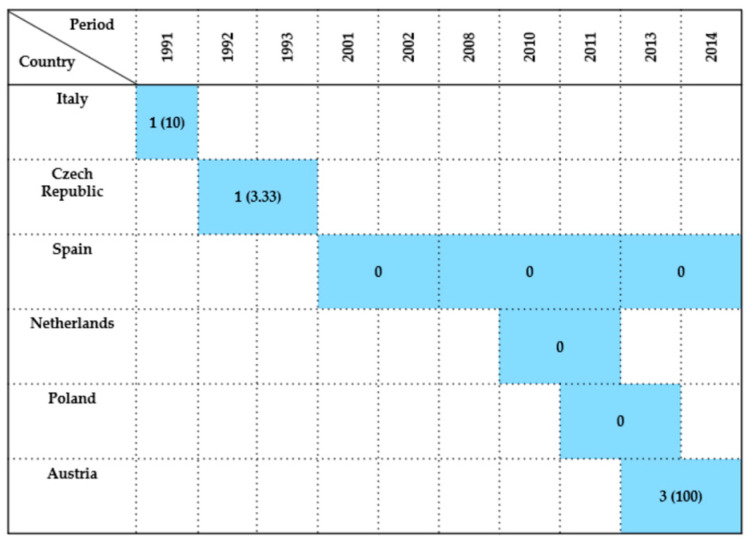
Visual representation of *Enterobacteriaceae* prevalence in *Gruiformes* by country and year. Colored cells represent positive findings; values in parentheses indicate the prevalence when available.

Overall, *Enterobacteriaceae* detection in *Gruiformes* was rare across most surveyed countries, with prevalence often at or near zero. The exception observed in Austria may reflect localized factors or sample size artifacts. Sampling relied mainly on cloacal swabs, while postmortem tissues and intestinal content were analyzed only occasionally. Studies originated primarily from Southern and Central Europe. The summarized data are graphically represented in Figure 10, highlighting inter-study and geographic variation.

#### 2.7.5. Charadriiformes

Studies investigating *Enterobacteriaceae* isolation in wild birds of the order *Charadriiformes* were conducted in Norway, Spain, Italy, and the Netherlands, covering a time span from 1969 to 2011.

In Norway, two separate studies provided contrasting data. Refsum et al. [17] detected one positive bird, in a common murre (*Uria aalge*), during a broad investigation from 1969 to 2000, although the total number of examined birds was not specified. In contrast, Kapperud & Rosef [48] found 0% prevalence among 76 birds sampled during 1980–1981. Later, Literak et al. [65] examined 215 little auks, but did not specified the results in this species.

In Spain, Millán et al. [21] tested four birds between 2001 and 2002 and reported 0% prevalence. Likewise, in Italy, Botti et al. [42] conducted a study from 2002 to 2010 that did not detect any *Enterobacteriaceae*-positive birds.

A more notable result emerged from the Netherlands, where Veldman et al. [44] reported between 2010 and 2011 a prevalence of 15.9%. This suggests a potentially higher circulation or detection rate of *Enterobacteriaceae* in *Charadriiformes* in that region and period.

**Figure 11:**
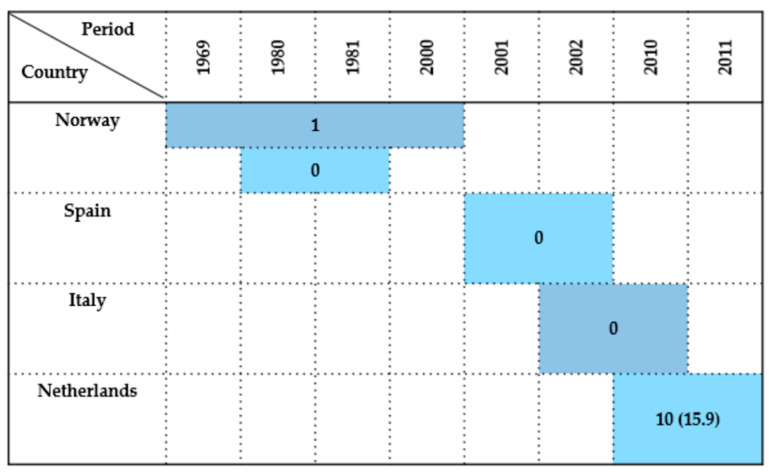
Visual representation of *Enterobacteriaceae* prevalence in *Charadriiformes* by country and year. Darker colored cells indicate positive findings with prevalence values in parentheses, while lighter colored cells indicate positive findings without prevalence data (number of isolates only).

In summary, the overall prevalence of *Enterobacteriaceae* in *Charadriiformes* was generally low across most regions, with studies from Northern and Southern Europe. However, the findings from the Netherlands contrast this trend and may reflect localized environmental factors, species-specific susceptibility, or methodological differences. Sampling relied primarily on cloacal swabs, while postmortem material and fecal samples were used only occasionally. A temporal and spatial overview of these results is presented in Figure 11.

#### 2.7.6. Other Seabird Orders

**Figure 12:**
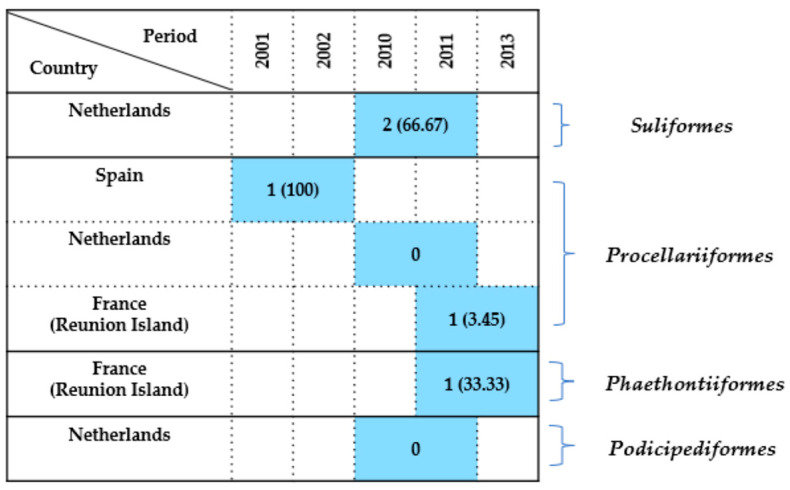
Detection rates of *Enterobacteriaceae* in wild seabirds belonging to the *Suliformes*, *Procellariiformes*, *Phaethontiiformes*, and *Podicipediformes* orders. Each box indicates the number of positive birds, followed by the prevalence in parentheses. Country and investigation period are shown alongside each record.

A limited number of studies (6) focused on less frequently investigated seabird orders, including *Suliformes*, *Procellariiformes*, *Phaethontiiformes*, and *Podicipediformes,* with the findings summarized in Figure 12.

Suliformes

A study conducted in the Netherlands between 2010 and 2011 reported a high prevalence of 66.67% (2/3) [44]. This may reflect localized environmental contamination or specific ecological conditions during the sampling period.

Procellariiformes

Data came from three countries. In Spain, Millán et al. [21] identified *Enterobacteriaceae* in the only individual sampled during 2001–2002, resulting in a prevalence of 100%, although from a single bird. On the other hand, in the Netherlands (2010–2011), Veldman et al. [44] tested three birds and found 0% prevalence. Meanwhile, Tessier et al. [59] reported 1 case among 29 *Procellariiformes* sampled on Réunion Island between 2011 and 2013, corresponding to a low prevalence of 3.45%.

Phaethontiiformes

The same study by Tessier et al. [59] recorded one case out of three individuals examined (33.3%) on Réunion Island during the 2011–2013 period, suggesting a moderate level of exposure or colonization in the *Phaethontiiformes* birds.

Podicipediformes

Only one study was available. Veldman et al. [44] found 0% prevalence among the two birds tested in the Netherlands during 2010–2011, indicating no detectable *Enterobacteriaceae* carriage in this small sample.

In conclusion, detection rates across these less frequently studied avian orders ranged from 0% to 100%, but interpretation remains limited due to small sample sizes and sparse geographic coverage. Positive detections originated mainly from Spain, France (Réunion Island), and the Netherlands, while other regions were not represented. Sampling relied primarily on cloacal swabs and postmortem tissues. Further research is needed to clarify the epidemiological relevance of *Enterobacteriaceae* in these seabird taxa.

### 2.8. Antimicrobial Resistance in Enterobacteriaceae Isolated from Wild Birds

A total of 41 studies conducted between 1995 and 2025 provided data on AMR profiles in *Enterobacteriaceae* isolated from wild birds across 18 European countries. These studies included a wide variety of avian orders, such as *Accipitriformes*, *Falconiformes*, *Anseriformes*, *Laridae*, *Columbiformes*, and others, from which were analyzed both cloacal swabs and fecal or postmortem samples [7,11,12,13,14,20,23,29,30,31,32,34,35,36,38,42,43,44,47,52,55,56,57,58,60,62,63,68,71,72,73,74,78,80,82,86,87,88,89,90].

The most frequently identified bacterial species were *Escherichia coli*, followed by *Salmonella enterica* and other genera, such as *Klebsiella*, *Enterobacter*, *Proteus*, *Citrobacter*, *Hafnia*, *Shigella*, and *Cronobacter*. Resistance profiles were tested against a broad spectrum of antimicrobials from over 15 different classes, including β-lactams (e.g., ampicillin, ceftazidime, cefotaxime), aminoglycosides (e.g., streptomycin, gentamicin), sulfonamides, tetracyclines, fluoroquinolones (e.g., ciprofloxacin, nalidixic acid), and polymyxins (e.g., colistin).

High rates of resistance were most consistently associated with several key antimicrobials. Ampicillin (AMP) emerged as the most frequently reported, with elevated resistance levels observed particularly in *E. coli* isolates from countries such as Spain [12,31,32,34,55,56,74], Poland [20,58,63,72,89], Switzerland [47,57], Sweden [23,71], and the Czech Republic [62,68,87]. Tetracycline (TET) also showed widespread resistance, identified in over 20 studies. This compound was often associated with co-resistance to sulfonamides, specifically sulfamethoxazole (SMX) [12,20,23,57,58], trimethoprim (TMP) [11,12,23,38,44,57,58,63,71,74,82], and a combination (SXT) [29,31,32,42,47,62,63,68,72,82,87,89], highlighting the occurrence of multi-drug resistance patterns.

Resistance to streptomycin (STR) and nalidixic acid (NAL) was frequently reported as well, especially among *Salmonella* and *E. coli* isolates obtained from *Laridae*, *Accipitriformes*, and *Anseriformes* [7,13,23,31,32,42,44,47,52,55,57,58,62,68,72,74,82]. These findings suggest consistent exposure to older classes of antibiotics in these avian hosts. Additionally, reduced susceptibility to third-generation cephalosporins, particularly cefotaxime (CTX) and ceftazidime (CAZ), was documented in multiple studies from France [90], the Netherlands [44], Spain [12,29,35,36,74,86], and the Czech Republic [68]. This is of particular concern due to the clinical relevance of extended-spectrum β-lactamase (ESBL)-producing strains, which may circulate between wildlife and human-related environments.

MDR phenotypes—defined as resistance to three or more antimicrobial classes—were documented in several studies, especially among gulls (*Laridae*) in France [13,60,90], Sweden [23,71], Spain [29,35,36,56,74], and the Netherlands [44]. For example, *E. coli* isolates from cloacal swabs of gulls in France (2016) showed resistance to all tested β-lactams [60], while a Spanish study (2019–2020) reported over 85% resistance to AMP, CTX, CAZ, and CIP among 87 *E. coli* isolates from *Accipitriformes* [12].

Rare or emerging species such as *Escherichia fergusonii*, *Shigella* spp., *Cronobacter sakazakii*, and *Hafnia alvei* were also identified with AMR traits, though less frequently. In several cases, isolates harbored resistance to carbapenems (e.g., imipenem, meropenem) and colistin, indicating the potential for zoonotic transmission of clinically relevant resistant strains.

Geographically, Spain, Poland, and Germany were among the most represented countries. Spain, in particular, reported the highest diversity of bird species, bacterial isolates, and antimicrobial agents tested.

The most commonly used sample types were cloacal swabs and feces, though several postmortem studies also contributed valuable data, especially regarding raptors.

A complete summary of the AMR data, including bacterial species, host species (order), country, antimicrobial agents tested, and resistance prevalence of their profiles, is provided in Table 1.

**Figure 13:**
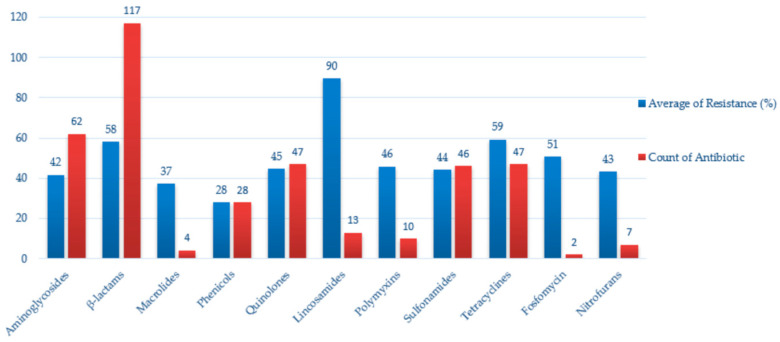
Average resistance percentages and reporting frequency for antimicrobial classes tested in *Enterobacteriaceae* isolates from wild birds in Europe. Blue bars represent the mean resistance values across all studies where the antimicrobial class was tested, while red bars indicate the number of records available per class.

As shown in Figure 13, resistance was most frequently reported against β-lactams, tetracyclines, and aminoglycosides, which were also the most commonly tested antimicrobial classes. High average resistance values were observed in lincosamides and tetracyclines, while sulfonamides and quinolones exhibited moderate levels. In contrast, nitrofurans and fosfomycin were less frequently and showed comparatively lower resistance.

These resistance patterns largely mirror antimicrobial usage trends in European human and veterinary medicine, where β-lactams, tetracyclines, and aminoglycosides remain among the most frequently applied classes. The overlap highlights the One Health concern that wild birds may act as environmental sentinels and potential reservoirs for resistance genes driven by anthropogenic antibiotic pressure.

## 3. Discussion

A limitation of this review is the uneven distribution of available studies across Europe. Spain accounted for the majority of investigations, particularly in *Accipitriformes*, *Strigiformes*, and *Pelecaniformes*, while other countries, such as Denmark, Ireland, and France (Réunion Island), were represented by single reports. This heterogeneity introduces a potential bias and indicates that some avian orders and regions are comparatively underrepresented.

**Figure 14:**
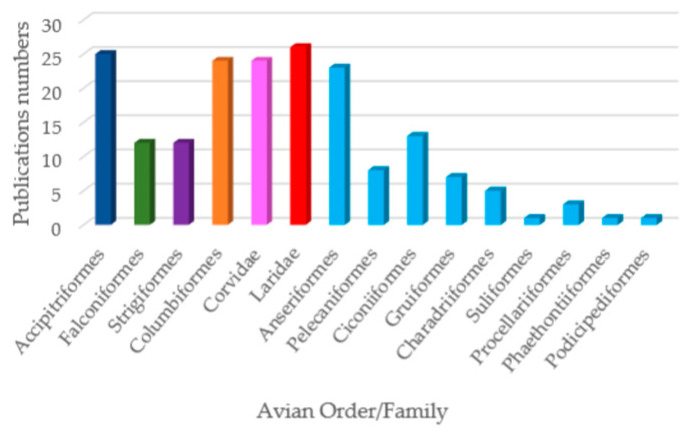
Distribution of *Enterobacteriaceae* studies from Europe across avian orders.

Within this uneven landscape, certain avian orders, such as *Accipitriformes*, *Laridae*, *Columbiformes*, and *Corvidae*, received the greatest research attention and are consistently reported as carriers of AMR *Enterobacteriaceae* (Figure 14). This focus likely reflects both ecological importance, as these species are often apex predators or synanthropic dwellers, and sampling bias, since they are more accessible to researchers through rehabilitation centers or urban environments.

**Figure 15:**
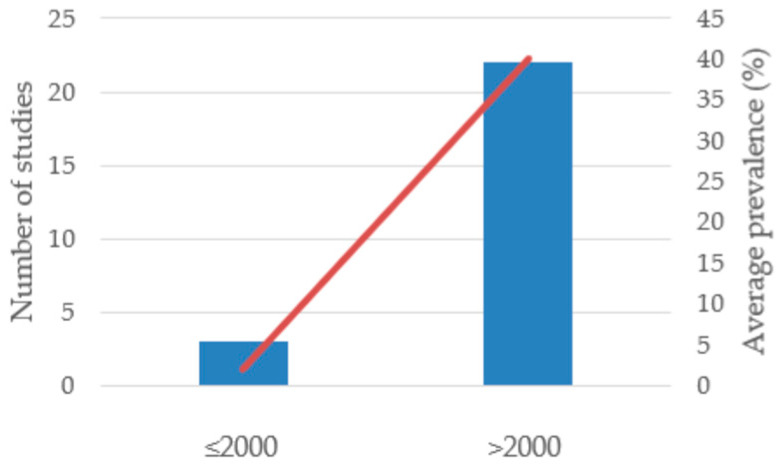
Temporal distribution of *Enterobacteriaceae* in *Accipitriformes* (red line shows the increasing trend).

*Accipitriformes* emerged as the most intensively studied order (Figure 15). This trend reflects growing research attention, improved diagnostic capacity, and recognition of raptors as key sentinels for antimicrobial resistance within One Health frameworks [12,28].

Early studies conducted before 2000 were few and often reported low or inconsistent prevalence, while post-2000 data indicate both higher detection rates and a wider geographical coverage. This temporal trend likely reflects increased research attention to raptors, improved diagnostic methods, and a growing recognition of their role as sentinels for AMR.

For instance, cloacal swabs collected from *Gyps fulvus* populations in Spain revealed high prevalence rates of *Salmonella enterica* (up to 52.6%), with serovars such as Typhimurium, Rissen, and Senftenberg being frequently isolated [28,32]. These findings are consistent with previous observations by Oteo et al. [35], who identified MDR *E. coli* and *Klebsiella pneumoniae* in raptors sampled during the 2015–2016 period.

Among falconiform birds, *Falco tinnunculus* and *F. peregrinus* were recurrently sampled across Spain and Italy. While *Salmonella* detection rates varied (0–20%), species such as *S. Enteritidis* and *S. Napoli* were recorded, sometimes alongside cephalosporin-resistant *E. coli* [31,41].

In owls (*Strigiformes*), both *Tyto alba* and *Strix aluco* showed colonization with clinically relevant *Enterobacteriaceae*, including ESBL-producing *E. coli* [29] and multiple *Salmonella* serovars such as Typhimurium and Enteritidis [42].

Birds from the order of *Columbiformes* (e.g., *Columba livia*) and the family of *Corvidae* (e.g., *Corvus frugilegus*, *C. corone*) were also frequently sampled in urban areas due to their synanthropic behavior. High isolation rates of *E. coli* were noted in pigeons across Germany, the Czech Republic, and France [14,46,60] with evidence of MDR [51].

Crows and magpies, on the other hand, harbored a broader spectrum of *Salmonella* serovars, including DT104 and DT41 [43,52].

Despite a taxonomic bias toward raptors and pigeons, several studies highlighted the presence of AMR *Enterobacteriaceae* in lessened bird taxa such as *Anseriformes* (e.g., *Anas platyrhynchos*, *Cygnus olor*) and *Pelecaniformes* (e.g., *Ardea cinerea*). Notably, *E. coli* isolates from mallards and swans frequently exhibited resistance to sulfonamides, tetracyclines, and third-generation cephalosporins [45,72].

Temporal analysis revealed that studies conducted after 2000 reported both a higher number of detections and greater prevalence values compared to earlier investigations. This increase likely reflects a combination of enhanced surveillance efforts, the wider application of microbiological methods, and a growing recognition of wild birds as reservoirs of AMR. Such differences highlight the importance of considering time-related bias when interpreting long-term patterns of *Enterobacteriaceae* occurrence and resistance.

The detection of resistant *Enterobacteriaceae* in wild birds also underscores potential transmission pathways at the wildlife–livestock–human interface. Migratory birds can disseminate resistant strains across countries and ecosystems, while synanthropic species feeding at landfills or agricultural areas may facilitate cross-contamination with domestic animals. These dynamics emphasize the One Health relevance of surveillance in wild bird populations, as they can act as both reservoirs and sentinels for AMR circulating in the environment.

Most detections of *Enterobacteriaceae* in wild birds appear to represent asymptomatic carriage rather than clinical disease, yet these isolates frequently show resistance to critically important antibiotics, underlining their epidemiological relevance. These patterns are consistent with the resistance trends summarized in the AMR section (Figure 13), where the highest values were observed for β-lactams, tetracyclines, and aminoglycosides.

However, heterogeneity in sampling design ranging from postmortem tissue recovery to cloacal swabs and fecal samples complicates direct comparisons across studies. Future studies should prioritize harmonized sampling and testing protocols, together with the integration of molecular approaches such as whole-genome sequencing. These methods would allow a deeper understanding of genetic relatedness between avian isolates and those from humans or livestock, thereby clarifying the epidemiological role of wild birds in the broader resistance network.

Taken together, these findings emphasize the uneven but consistent detection of resistant *Enterobacteriaceae* across diverse avian taxa, reinforcing the importance of harmonized surveillance under a One Health framework.

## 4. Materials and Methods

This systematic literature review was conducted in accordance with PRISMA 2020 guidelines (Page et al., 2021) [91], aiming to synthesize data on the occurrence and AMR of *Enterobacteriaceae* in mostly free-ranging wild birds across Europe.

Eligible studies were those that investigated wild bird populations sampled in European countries and reported data on the isolation and identification of *Enterobacteriaceae*. To be included, studies had to provide sufficient methodological details such as the country of sampling, investigation period, bird species or taxonomic group, sample type, and number of positive samples or isolates. For studies reporting AMR, inclusion required details on the antimicrobial agents tested and the corresponding resistance data.

Studies were excluded if they focused only on captive or domestic birds, lacked geographic or taxonomic information, were not published in English, or did not present data about the *Enterobacteriaceae* bacterial family. All eligible studies were categorized based on avian taxonomic group (order or family), country of sampling, sampling period, and availability of AMR data.

The literature search was conducted using the PubMed database https://pubmed.ncbi.nlm.nih.gov (accessed on 1 July 2025), applying the search query (*Enterobacteriaceae*) AND (wild birds) AND (Europe). Filters were applied for language (English), and the publication date range included all records from 1971 to 2025; although the review covered 1969–2025, PubMed records began in 1971. The last search was completed on 1 July 2025, and a total of 147 records were retrieved. Additional articles were identified by reviewing the reference lists of relevant studies.

In addition to the PubMed search, a supplementary search was also conducted in the Web of Science database https://www.webofscience.com (accessed on 1 July 2025) using the same search strategy. This search identified ten additional studies, of which four had already been included through the initial search; one had previously been excluded, two were removed, as they focused on ungulate species; one was not geographically relevant (conducted outside Europe); and two were retained for further assessment.

Finally, results for all articles were imported into a bibliographic referencing tool (Zotero Desktop 6.0.36). Following removal of duplicates, the titles and abstracts of 145 articles were screened for relevance. After this screening, 113 articles were retained for full-text review. The full texts were then assessed for eligibility, resulting in 80 studies included in the analysis of *Enterobacteriaceae* occurrence, and 41 studies retained for AMR data synthesis.

**Figure 16:**
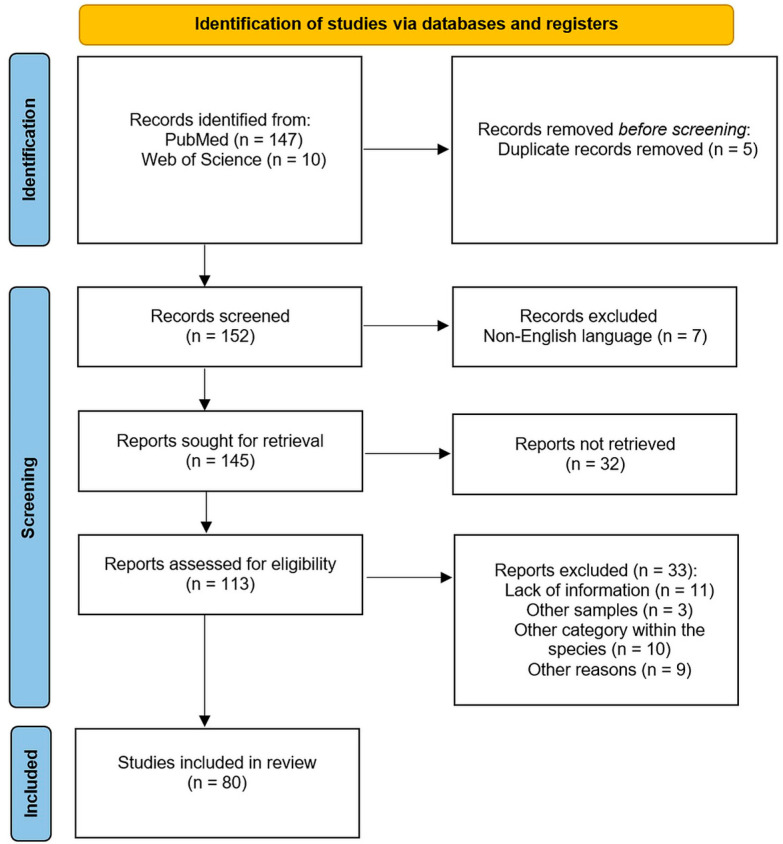
Identification of studies regarding the occurrence of *Enterobacteriaceae* bacterial family and AMR in wild birds via databases using PRISMA guidelines.

All screening and selection steps were performed manually by the lead reviewer and two other reviewers, with a fourth reviewer consulted in case of uncertainty. No attempts were made to contact the study authors for data confirmation, as all required information was available from the reports. Data extraction was conducted manually without the use of automation tools. The study selection process is illustrated in Figure 16, following the PRISMA 2020 flowchart template, which outlines the number of records identified, screened, excluded, and included in the final review.

Data from the studies included were extracted into structured tables created in Microsoft Excel. Extracted variables included the country of origin, investigation period, wild bird species examined, sample type, number of positive samples and percentage, number of birds examined or isolates recovered, antimicrobials tested (when applicable), resistance results, and full reference details.

Birds were grouped taxonomically by order or family, including *Accipitriformes*, *Falconiformes*, *Strigiformes*, *Corvidae*, *Columbiformes*, *Anseriformes*, *Pelecaniformes*, *Ciconiiformes*, *Gruiformes*, *Charadriiformes*, *Laridae*, *Suliformes*, *Procellariiformes*, *Phaethontiiformes*, and *Podicipediformes*. All data were manually extracted and verified for consistency and completeness.

The primary outcomes of interest were the detection of *Enterobacteriaceae* in wild bird samples, expressed as the number and percentage of positive birds or isolates, and the presence of antimicrobial resistance, expressed as the number of isolates resistant to specific antimicrobials or classes.

Additional contextual data, such as bird species and sampling methodology, were collected when available. If species-level identification or isolated counts were unclear, the data were reported as stated in the original source, and any missing values were noted accordingly. Throughout the manuscript, “prevalence” is used when both numerator and denominator were available, while “detection” refers to reports without a denominator.

All extracted data were reviewed for consistency, and no assumptions were made beyond the information provided in the original publications. Further information is available in Supplementary Table S1.

## 5. Conclusions

This systematic review demonstrates that wild birds across Europe harbor *Enterobacteriaceae*, often resistant to multiple antibiotics of clinical relevance. The highest prevalence was reported in Southern and Central Europe, while Northern and Eastern regions remain underrepresented. Resistance was particularly frequent against broad-spectrum β-lactams, tetracyclines, and fluoroquinolones, underscoring the role of wild birds as potential reservoirs and sentinels for AMR. The heterogeneity of sampling methods and the uneven geographical coverage highlight the need for harmonized surveillance and integration of molecular approaches, such as whole-genome sequencing, within a One Health framework.

## Figures and Tables

**Table 1:** Summary of antimicrobial resistance in *Enterobacteriaceae* isolated from wild birds in Europe (1995–2025). Data include country, sample type, period of investigation, avian species, bacterial species (number of isolates), antimicrobials tested, and resistance prevalence findings.

Country	Sample Type	Period	Wildlife Species Examined	Species (No. of Isolates)	Antimicrobials Tested	Resistance (%)	References
UK	Post-mortem	1995–2003	*Accipitriformes*	*Salmonella enterica serovar* Typhimurium *DT104* (1)	n.a.	AMP, CHL, STR, S, TET-(100)	Pennycott et al. [43]
France	Cloacal swabs	2009	*Laridae*	*Escherichia coli* (153)	TET, AMP, STR, CHL, NAL, CFR	TET (39), AMP (25), STR (19), CHL (7), NAL (3), CFR (3)	Bonnedahl et al. [13]
Sweden	Cloacal swabs	2008	*Laridae*	*Escherichia coli* (83)	TET, AMP, STR, CHL, NAL, CFR, SMX, FOS, TGC, TMP, NIT, MEC	TMP (2.4), STR (2.4), CHL (1.2), NIT (1.2), CFR (1.2), FOS (1.2)	Bonnedahl et al. [71]
Germany	Cloacal swabs/post-mortem	2006/2008	*Accipitriformes* *Falconiformes* *Strigiformes* *Columbiformes* *Laridae* *Corvidae*	*Escherichia coli* (188)	AMP, CHL, GEN, SP, STR, TET	AMP (7.45), CHL (1.6), GEN (2), SP (8.5), STR (10), TET (8.5)	Guenther et al. [14]
Poland	Cloacal swabs/feces	2008–2009	*Anseriformes* *Laridae*	*Escherichia coli* (31)	AMC, AMP, KF, CIP, CHL, GEN, NAL, STR, S, SXT, TET	AMP (48.4), GEN (9.7), NAL (38.7), STR (67.7), S (16), TET (22.6), AMC (9.7), KF (13), CIP (13), SXT (13), CHL (3.2)	Literak et al. [72]
Norway	Feces	2010	*Laridae*	*Enterobacter cloacae* (2)	AMP, SAM, CFZ, CXM, FOX, GEN, SXT, COL, OXA, OFX, TET, ATM, PIP, TZP, CFP, CTX, CAZ, FEP, SCF, MER, CIP, TGC, TOB, AMK	AMP, CFZ, FOX-(100)	Literak et al. [65]
Belgium	Cloacal swabs	2011	*Anseriformes*	*Escherichia coli* (2)	AMP, CEF, AMC	CEF (100)	Garmyn et al. [80]
Spain	Feces	2009–2011	*Columbiformes* *Passeriformes*	*Salmonella enterica serovar* Typhimurium (15)	NAL, CIP, CTX, AMP, CHL, STR, GEN, SFX, TMP, TET	AMP (20), STR (20), S (13.33), TET (20), NAL (6.66), CHL (6.66)	Andrés et al. [55]
Italy	Cloacal swabs	2002–2010	*Strigidae*	*Salmonella enterica* *serovar* Typhimurium *DT 193*	AMP, AMC, CTX, KF, OXY, TET, AMK, STR, NEO, GEN, K, NAL, ENR, CIP, CHL, SXT, COL	AMP, AMC, NEO, STR, OXY, TET-(100)	Botti et al. [42]
			*Accipitridae*	*Salmonella enterica* *serovar* Typhimurium		AMP, AMC, STR, OXY, TET-(100)	
			*Falconidae*	*Salmonella enterica* *serovar* Brancaster		AMP, AMC, NAL, NEO, STR, K, OXY, TET, SXT-(100)	
			*Accipitridae*	*Salmonella enterica* *serovar* Ohio		AMP, AMC, STR, OXY, TET-(100)	
Germany	Cloacal swabs	2013	*Accipitriformes*	*Salmonella enterica* subsp. *enterica serovar* Corvallis	IPM, ERT, MER, CHL, K, TET, TMP, STR, S, FOS	IPM, CHL, K, TET, TMP, STR, S, FOS-(100)	Fischer et al. [38]
The Netherlands	Cloacal swabs/postmortem	2010–2011	*Accipitriformes* *Falconiformes* *Columbiformes* *Anseriformes* *Pelecaniformes* *Ciconiiformes* *Gruiformes* *Charadriiformes* *Suliformes* *Procellariiformes* *Laridae* *Corvidae* *Sturnidae*	*Escherichia coli* (65)	AMP, CTX, CAZ, CIP, CHL, FFC, GEN, K, NAL, STR, SMX, TMP, TET	AMP (100), CEF (100), CAZ (97), CHL (34), CIP (48), FFN (6), GEN (23), NAL (46), STR (57), SUL (66), TET (61.5), TMP (58.5), K (38.5)	Veldman et al. [44]
Switzerland	Cloacal swabs	2011–2012	*Columbiformes* *Ciconiiformes*	*Escherichia coli* (6)	AMP, KF, CTX, FEP, IPM, AMC, NAL, CIP, STR, TET, SMX, TMP, CHL	AMP (100), KF (100), CTX (50), AMC (50), NAL (66.66), CIP (16.66), STR (66.66), TET (100), SMX (50), TMP (50)	Zurfluh et al. [57]
Ireland	Feces	2013	*Laridae* *Sturnidae*	*Escherichia coli* (115)	AMC, AMP, CIP, STR, TET, PG, MER	TET (8.7), STR (6), AMP(1.7)	Carroll et al. [73]
Czech Republic	Feces	2010–2013	*Corvidae*	*S. Typhimurium* (2) *S. Hadar* (1)	AMC, AMP, KF, CAZ, CHL, CIP, GEN, NAL, STR, SXT, S, TET	AMP (33.33), CHL (33.33), STR (66.66), TET (66.66), NAL (33.33), SXT (33.33)	Janecko et al. [62]
France	Feces	2011		*S. Montevideo* (2)		TET (100)	
Germany	Feces	2011		*S. Senftenberg* (1)		KF (100)	
Poland	Feces	2011		*S. Enteritidis* (5)		NAL (60)	
Slovakia	Feces	2013		*S. Infantis* (4)		NAL, SXT, TET-(100)	
Spain	Feces	2011		*S. Oranienburg* (2)		SXT (50), NAL (50), S (50)	
Spain	Cloacal swabs	2013–2014	*Acipitriformes* *Falconiformes* *Strigiformes*	*S.* Typhimurium monophasic 4,12:i:- (6) *S.* Hadar 6,8:z10:e,n,x (1) *S.* Enteritidis 9,12:g,m:- (1)	AMP, AMC, APR, CHL, CEF, COL, FFC, FL, GEN, NAL, NEO, STR, SXT, S, TET	AMP (75), AMC (12.5), CHL (37.5), STR (62.5), S (62.5), SXT (37.5), CEF (12.5), GEN (37.5), COL (50), APR (37.5), TET (37.5), FL (12.5), FFC (12.5)	Molina-López et al. [31]
Germany	Feces	2007–2011	*Anseriformes*	*Escherichia coli* (400)	AMK, AMC, AMP, SAM, CZO, CTX, CAZ, FOX, CXM, CHL, CIP, DOX, K, GEN, IPM, LEV, MER, NEO, TZP, STR, S3, SXT, TET, TIC, TOB, TMP	AMC (0.75), AMP (5.75), SAM (2.25), CZO (0.25), FOX (0.25), CXM (0.25), CHL (1.75), CIP (0.75), DOX (4), K (0.5), GEN (0.75), LEV (0.5), NEO (0.25) TZP (0.25), STR (4), S3 (3.25), SXT (1), TET (4.5) TIC (5), TMP (2)	Rödiger et al. [82]
Spain	Cloacal swabs	2013–2014	*Accipitriformes* *Falconiformes* *Strigiformes* *Columbiformes* *Anseriformes* *Pelecaniformes* *Ciconiiformes* *Gruiformes* *Laridae* *Sturnidae*	*Escherichia coli* (16)	AMP, AMC, CTX, CAZ, CRO, FOX, IPM, NAL, CIP, GEN, AMK, TOB, CHL, SXT, TET	CHL (50), NAL (87.5), CIP (75), GEN (25), TOB (25), TET (75), SXT (44), AMC (12.5), FOX (12.5), CAZ (69), CTX (100)	Alcalá et al. [29]
Spain	Cloacal swabs	2013	*Ciconiiformes*	*Escherichia coli* (104)	GEN, CTX, ENR	GEN (47), CTX (27), ENR (41.3)	Camacho et al. [86]
Spain	Feces	2012–2014	*Accipitriformes* *Falconiformes* *Strigiformes* *Anseriformes*	*Salmonella* serovars Anatum (1), Bredeney (1), Enteritidis (4), London (1), Mikawasima (1), *Salmonella* spp. (2), Typhimurium (5)	TET, GEN, CIP, ERY	ERY (93.33), TET (60)	Jurado-Tarifa et al. [30]
Poland	Cloacal swabs	2011–2013	*Anseriformes* *Ciconiiformes*	*Escherichia coli* (96)	AMX, ENR, TET	AMX (19.8), ENR (2), TET (8.33)	Kuczkowski et al. [78]
The Netherlands			*Anseriformes*	*Escherichia coli* (94)		AMX (24.5), TET (1)	
Italy	Post-mortem	2010–2013	*Corvidae*	*S. Bredeney*	AMC, CIP, CTX, SXT, CHL, AMP, STR, TET, GEN, NAL, COL, KF	AMP, STR, TET, NAL-(100)	Rubini et al. [52]
				*S. Enteritidis*		AMP (100)	
				*S. Typhimurium* (5)		AMP (60), STR (20), COL (40), KF (20)	
Sweden	Feces	2013	*Laridae*	*Escherichia coli* (29)	AMP, CIP, NAL, GEN, STR, TET, FFC, COL, SMX, TMP, CHL, K, CTX, CAZ, FOX, TOB, TZP, AMC AMC, TGC, NIT, MER, AMK, AMK, ERT, IPM, FOS	AMC (72.4), AMP (100), CTX (100), CAZ (65.5), TET (62), STR (31), TMP (48.3), SMX (44.8), FFC (7), CIP (69), NAL (55.2), GEN (34.5), CHL (20.7), K (13.8), TOB (17.2), TZP (7)	Atterby et al. [23]
Poland	n.a.	2011–2014	*Columbiformes* *Anseriformes* *Ciconiiformes* *Passeriformes (Turdidae)* *Apodidae*	*Salmonella* spp. (36)	SMX, GEN, STR, K, CIP, NAL, CTX, CAZ, AMP, TET, FFC, CHL, COL, TMP	SMX (94.44), CIP (8.33), NAL (8.33), COL (22.22), TMP (8.33), TET (8.33), AMP (5.55), STR (11.11), CHL (5.55), FFC (11.11), K (5.55)	Krawiec et al. [58]
Spain	Feces	2016	*Accipitriformes*	*Salmonella enterica* (38) serotypes Typhimurium monophasic 4,12:i:- (1), Typhimurium 4,12:i:1,2 (1)	CIP, CAZ, AMP, AMX, AMC, STR, GEN, NEO, SXT, TET, DOX	AMP (75), AMX (75), AMC (10), STR (77.5), GEN (30), NEO (65), SXT (10), TET (70), DOX (77.5)	Blanco et al. [32]
Spain	Cloacal swabs	2015–2016	*Accipitriformes* *Falconiformes* *Strigiformes* *Laridae* *Corvidae*	*Escherichia coli* (60), *Klebsiella pneumoniae* (10), *Hafnia alvei* (10), *Enterobacter* spp. (8), *Proteus mirabilis* (4), *Citrobacter freundii* (1), *Morganella morganii* (1)	CTX, AMC, FOX, CLX	CTX (100)	Oteo et al. [35]
Slovak Republic	Feces	2017	*Accipitriformes*	*Escherichia coli* (19)	AMP, SAM, ERT, CEF, CRO, CAZ, CAC, CFQ, GEN, STR, NEO, SPE, NAL, ENR, CIP, CHL, FFC, TET, COL, SXT, COT	AMP (52.6), TET (52.6), NAL (42.1), STR (26.3), ENR (21), CIP (21), COT (21)	Handrova and Kmet [7]
Czech Republic	Cloacal swabs	2010–2013	*Phasianidae*	*Escherichia coli* (180)	AMC, AMP, CEF, SCF, CIP, COL, GEN, CHL, NEO, TZP, STR, SXT, TET	AMP (72.22), CEF (89), CHL (5.55), SXT (5.55), TET (22.22)	Holko et al. [87]
Spain	Feces/cloacal swabs	2015–2016	*Accipitriformes*	*S. Enteritidis* (4)	AMP, CTX, CAZ, GEN, NAL, CIP, COL, CHL, AZM, TGC, SXT, TMP	AMP (50)	Martín-Maldonado et al. [34]
				*S. Typhimurium* (4)		AMP (50), TGC (25)	
				*S. Houston* (4)		AMP (25)	
				*S. Manhattan* (1)		AMP (100)	
				*S. Schleissheim* (1)		AMP (100)	
France	Feces	2016	*Laridae* *Columbifomes*	*Escherichia coli* (5)	AMX, AMC, KF, CRO, FEP, TZP, ERT, IPM, FOS, NIT, SXT, AMK, CIP, COL, GEN	AMX (80), AMC (60), CRO (80), FEP (80), STX (40), CIP (20), KF (20)	Ngaiganam et al. [60]
				*Cronobacter sakazakii* (1)		AMX, AMC, CRO, FFC-(100)	
				*Hafnia alvei* (8)		AMX (4), AMC (4), KF (6), COL (6), FFC (2), NIT (2)	
				*Proteus hauseri* (1)		NIT, COL-(100)	
				*Panteoa ananatis* (1)		AMX, AMC, KF, FFC, NIT, COL-(100)	
				*Serratia marcescens* (1)		NIT, COL-(100)	
UK	Feces	2016	*Passeriformes* *Columbiformes*	*Escherichia coli* (27)	AMP, CPD, COL, APR, IPM, TMP, TET, CIP	AMP (51.9), CEF (26), COL (18.5), APR (11.11), IPM (11.11), TMP (18.5), TET (22.22), CIP (11.11)	Swift et al. [11]
Switzerland	Cloacal swabs	2018	*Accipitriformes* *Anseriformes* *Falconiformes* *Columbiformes* *Laridae* *Passeriformes* *Corvidae*	*Escherichia coli* (256)	AMP, AMC, AZM, CHL, CIP, CZS, NIT, GEN, K, NAL, STR, SXT, TET, CTX, FEP	AMP (5), NAL (4), TET (5.5), CHL (2.3), CIP (2.3), CZS (3.5), STR (2.3), SXT (3.1), AMC (0.4), AZM (1.2), NIT (0.4), GEN (1.2), K (1.6), FEP (0.4), CTX (2)	Zurfluh et al. [47]
Slovacia	Contents of the appendix	2020	*Phasianidae*	*Escherichia coli* (70)	AMP, TET, CTX, CAZ	CTX (100), CAZ (100), AMP (100), TET (87)	Hleba et al. [88]
Spain	Cloacal swabs	2018–2019	*Columbiformes* *Ciconiiformes* *Laridae* *Passeriformes* *Sturnidae*	*Salmonella* spp. (37)	CIP, NAL, AMP, FOX, CAZ, MER, CHL, SMX, COL, AZM, TGC, GEN, TMP, TET	CIP (30), NAL (30), AMP (10.8), COL (21.6), TET (13.5)	Martín-Maldonado et al. [56]
Poland	Cloacal swabs	2017	*Accipitriformes* *Falconiformes* *Anseriformes* *Passeriformes* *Strigiformes*	*Escherichia coli* (32)	TET, GEN, K, CIP, AMP, CHL, SXT	TET (50), GEN (34.4), CIP (47), AMP (28), K (18.75), CHL (6.25), SXT (34.4)	Nowaczek et al. [89]
Spain	Buffers on the bone surface of fractures	2019	*Accipitriformes*, *Falconiformes*, *Charadriiformes*, *Strigiformes*, *Ciconiiformes*, *Pelecaniformes*, *Apodiformes*, *Passeriformes*	*Escherichia fergusonii* (9)	CL, CZS, CEF, ENR, CTX	CL (77.8), CZS (33.33)	Tardón et al. [36]
				*Escherichia marmotae* (1)		CL (100)	
				*Enterobacter cloacae* (1)		CL, CZS-(100)	
				*Enterobacter kobei* (1)		CL (100)	
				*Enterobacter ludwigii* (1)		CL, CZS-(100)	
				*Enterobacter faecalis* (4)		CL (50), CZS (100), CEF (100), CTX (100)	
				*Hafnia alvei* (2)		CL, CZS-(100)	
				*Leclercia adecarboxylata* (1)		CL (100)	
				*Pantoea agglomerans* (5)		CL (60)	
				*Proteus mirabilis* (1)		CL (100)	
				*Shigella boydii* (1)		CL (100)	
				*Shigella flexneri* (5)		CL (80)	
				*Shigella sonnei* (1)		CL (100)	
France	Cloacal swabs	2016	*Laridae*	*Escherichia coli* (51) *Enterobacter cloacae* (1) *Klebsiella pneumoniae* (4) *Proteus mirabilis* (2) *Citrobacter freundii* (1) *Enterobacter kobei* (1) *Escherichia albertii* (1) *Escherichia fergusonii* (1) *Hafnia alvei* (1) *Klebsiella aerogenes* (1)	CTX, CAZ	CTX, CAZ-(100)	Vittecoq et al. [90]
Spain	Cloacal swabs	2019–2020	*Accipitriformes*	*Escherichia coli* (87)	AMP, CTX, CAZ, MER, NAL, CIP, GEN, TET, TGC, AZM, CHL, COL, SMX, TMP	AMP (100), CTX (100), CAZ (100), CIP (95.4), TET (87.35), SMX (85), TMP (84), NAL (71.3), CHL (69), GEN (41.4), AZM (35.6)	Guitart-Matas et al. [12]
Poland	Feces	2017–2018	*Phasianidae*	*Escherichia coli* (27)	AMC, CIP, TET, SMX, GEN, AMP, NAL, COL	AMC (7.4), SMX (52)	Kwaśna et al. [20]
Poland	Cloacal swabs	n.a.	*Corvidae*	*Escherichia coli* (31)	AMP, AMC, CTX, MER, IPM, GEN, K, STR, TET, CIP, S, SXT, TMP, CHL, CTX/C	TET (29), AMP (26), AMC (3.2), S (13), TMP (13), SXT (13), CHL (6.45)	Łopucki et al. [63]
Czech Republic	Cloacal swabs	2018–2019	*Laridae*	*Escherichia coli* (141)	AMP, STR, S, S3, TET, SXT, CHL, CZS, NAL, CAZ, CAZ, GEN, AMC, CIP, ERT, IPM, ATM, NIT, AZM, COL	AMP (89.4), STR (18), S (25), TET (32), SXT (17), CHL (10.6), CZS (61), NAL (31.2), CAZ (49.6), GEN (34), AMC (59), CIP (23), ERT (33), ATM (61), NIT (0.7), AZM (19), COL (0.7)	Nesporova et al. [68]
Spain	Cloacal swabs	2009–2018	*Laridae*	*Salmonella* Typhimurium and monophasic *S.* Typhimurium (Typhimurium m.) (23)	AMP, AZM, CTX, TAZ, CHL, NAL, CIP, COL, GEN, MER, TET, TGC, SMX, TMP	AMP (39), NAL (4.3), CIP (4.3), TET (26), TMP (8.7)	Manzanares-Pedrosa et al. [74]
				*Bredeney* (4)		AMP (50), CTX (50), TAZ (50), CHL (50), TET (100), TMP (50)	
				*Infantis* (2)		NAL, CIP, TMP, TET (100)	
				*London*		TMP, TET-(100)	
				*Mons*		NAL, CIP-(100)	
				*Virchow*		NAL, CIP, TMP, TET-(100)	

AMC—amoxicillin/clavulanate, AMX—amoxicillin, AMK—amikacin, AMP—ampicillin ATM—aztreonam, AZM—azithromycin, APR—apramycin, CAZ—ceftazidime, CEF—ceftiofur, CHL—chloramphenicol, CIP—ciprofloxacin, COL—colistin, CRO—ceftriaxone, CTX—cefotaxime, CZO—cefazoline, CZS—cefazolin, CPD—cefpodoxime, CXM—cefuroxime, COT—cotrimoxazole, CAC—ceftazidime/clavulanate, CFQ—cefquinome, CL—clindamycin, CFP—cefoperazone, CFR—cefadroxil, CPD/C—cefpodoxime/clavulanic, CLX—cloxacillin, CTX/C—cefotaxime/clavulanic, DOX—doxycycline, ENR—enrofloxacin, ERT—ertapenem, ERY—erythromycin, FEP—cefepime, FFC—florfenicol, FOS—fosfomycin, FOX—cefoxitin, FL—flumequine, GEN—gentamicin, IPM—imipenem, K—kanamycin, KF—cephalothin, LEV—levofloxacin, MER—meropenem, MEC—mecillinam, NAL—nalidixic acid, NIT—nitrofurantoin, NEO—neomycin, NOR—norfloxacin, OXA—oxolinic acid, OXY—oxytetracycline, OFX—ofloxacin, PIP—piperacillin, PG—penicillin G, S—sulfonamide, STR—streptomycin, SMX—sulfamethoxazole, SXT—sulfamethoxazole/trimethoprim, SAM—ampicillin/sulbactam, SP—spectinomycin, S3—compound sulfonamides, SPE--spectinomycin, SCF—cefoperazone/sulbactam, SFX—sulfisoxazole, TET—tetracycline, TGC—tigecycline, TMP—trimethoprim, TOB—tobramycin, TZP—piperacillin/tazobactam, TIC—ticarcillin, TAZ—tazobactam, n.a.—not available.

## Data Availability

All data are included within the article.

## Supplementary Materials

The following supporting information can be downloaded at: https://www.mdpi.com/article/10.3390/antibiotics14090905/s1, Table S1: Wildlife species examined and bacterial species identified per year and country.
