# Supplementary material for: Prevalence and Antimicrobial Resistance of Enterobacteriaceae in Wild Birds Across Europe: A Systematic Review
Source: Antibiotics (Basel). 2025 Sep 8;14(9):905. doi: 10.3390/antibiotics14090905 (PMC12466876; doi:10.3390/antibiotics14090905)
Supplement: Supplementary file 1 [file antibiotics-14-00905-s001.zip › antibiotics-3813900-supplementary.pdf]

Table S1. Wildlife species examined and bacterial species identified per year and country

Country	Investigation Period	Wildlife Species Examined	Sample Type	No.of Positive (%)	No.of Examined	Species (No.of Isolates)	Reference
		Accipitriformes					
Norway	1969–2000	Eurasian Sparrowhawk (*Accipiter nisus*)<br>Osprey (*Pandion haliaetus*)	postmortem	**3**	**NA**	*Salmonella enterica* serovar *Typhimurium* O:4,12 (2) and O:4,5,12 (1)	Refsum et al. [17]
Denmark	2001-2002	Buzzard (*Buteo buteo*)	cloacal swabs	**0 (0)**	**2**	*Salmonella* spp.	Skov et al. [26]
Spain	3 years period	Sparrowhawk (*Accipiter nisus*)<br>Goshawk (*Accipiter gentilis*)<br>Montagu's harrier (*Circus pygargus*)<br>Buzzard (*Buteo buteo*)<br>Imperial eagle (*Aquila heliaca*)<br>Black vulture (*Aegytius monachus*)<br>Griffon vulture (*Gyps fulvus*)	faeces/<br>cloacal swabs	**10 (5.7)**	**176**	*Salmonella* serotype Havana (5), Adelaide (2), Brandenburg (1), Typhimurium DT104b (1), Virchow PT8 (1)	Reche et al. [27]
	2001-2002	Griffon vulture (*Gyps fulvus*)<br>Sparrowhawk (*Accipiter nisus*)<br>Buzzard (*Buteo buteo*)<br>Harris's hawk (*Parabuteo unicinctus*)<br>Red kite (*Milvus milvus*)<br>Black kite (*Milvus migrans*)<br>Goshawk (*Accipiter gentilis*)<br>Honey buzzard (*Pernis apivorus*)	postmortem	**4 (13.34)**	**30**	*Salmonella* Typhimurium (2)<br>*S*. Muenchen (1)<br>*Salmonella* spp. (1) not serotyped	Millán et al. [21]
	2013	Wild Griffon Vultures (*Gyps fulvus*)	cloacal swabs	**51 (52.6)**	**97**	*Salmonella enterica* subsp. *enterica* (49)<br>Serotypes: Typhimurium (42), Rissen (4), Senftenberg (3) and 4,12:b[-] (2).<br>*Salmonella enterica* subsp. *salamae* (2)	Marin et. al. [28]
	2013-2014	Egyptian vulture (*Neophron percnopterus*)<br>Eurasian griffon Vulture (*Gyps fulvus*)	cloacal swabs	**2 (100)**	**2**	*S. enterica* subsp. *enterica* serovar Typhimurium (2)	Molina-López et al. [31]

2013-2014	Black kite (*Milvus migrans*), Red kite (*Milvus milvus*), Golden eagle (*Aquila chrysaetos*),<br>Common buzzard (*Buteo buteo*),<br>Booted eagle (*Hieraaetus pennatus*)<br>Short-toed snake eagle (*Circaetus gallicus*)<br>Eurasian sparrowhawk (*Accipiter nisus*)<br>Northern goshawk (*Accipiter gentilis*)<br>Griffon vulture (*Gyps fulvus*)<br>Bearded vulture (*Gypaetus barbatus*)<br>Western marsh harrier (*Circus aeruginosus*)	cloacal swabs	**5 (10.2)**	**49**	*Escherichia coli*	Alcalá et. al. [29]
2012-2014	Booted eagle (*Hieraaetus pennatus*)<br>Short-toed snake Eagle (*Circaetus gallicus*)<br>Golden eagle (*Aquila chrysaetos*)<br>Griffon vulture (*Gyps fulvus*)<br>Short-toed snake Eagle (*Circaetus gallicus*)<br>Black-winged kite (*Elanus caeruleus*)<br>Sparrowhawk (*Accipiter nisus*)<br>Black kite (*Milvus nigrans*)<br>Red kite (*Milvus milvus*)<br>Common buzzard (*Buteo buteo*)<br>Montargu's harrier (*Circus pygargus*)<br>Western marsh harrier (*Circus aeruginosus*)<br>European honey buzzard (*Pernis apivorus*)	faeces	**4 (4.82)**	**83**	*Salmonella* Typhimurium (3), *Salmonella* spp. (1)	Jurado-Tarifa et. al. [30]
2016	Egyptian vultures (*Neophron percnopterus*)<br>Griffon vultures (*Gyps fulvus*)	faeces	**39 (34.2)**	**114**	*Salmonella enterica*<br>Typhimurium 4,12:i:1,2 (9), Typhimurium monophasic 4,12:i- (26), Schwarzengrund 4,12:d:1,7 (1), Israel 9,12:e,h:e,n,z15 (1) and S. diarizonae 61:1,k:1,5,7 (2)	Blanco et. al. [32]
2016	Griffons (*Gyps fulvus*)	cloacal swabs	**22 (21.1)**	**104**	*Salmonella* spp. *Salmonella* Typhimurium monophase 4,12:i:-	Marin et al. [33]
2015 - 2016	Common buzzard (*Buteo buteo*)<br>Booted eagle (*Hieraaetus pennatus*)<br>Griffon vulture (*Gyps fulvus*)<br>Black vulture (*Aegytius monachus*)<br>Bonelli's eagle (*Aquila fasciata*)<br>Golden eagle (*Aquila chrysaetos*)<br>Eurasian sparrowhawk (*Accipiter nisus*)	cloacal swabs	**39**	**NA**	*Escherichia coli* (24), *Klebsiella pneumoniae* (4), *Hafnia alvei* (7), *Proteus mirabilis* (1)<br>*Enterobacter* spp. (3)	Oteo et al. [35]

		Northern goshawk (*Accipiter gentilis*)<br>European honey buzzard (*Pernis apivorus*)<br>Black kite (*Milvus nigrans*)					
	2015-2016	Wild Bonelli's eagle (*Aquila fasciata*)	faeces	**28 (45.2)**	**62**	*Salmonella* spp. (28)<br>Serovars: Enteritidis (4), Typhimurium (4), Houston (4), Cerro (3), Manhattan (1), Carnac (1), Tomegbe (1), Schleissheim (1)	Martín-Maldonado et al. [34]
	2019	Goshawk (*Accipiter gentilis*)<br>Western marsh harrier (*Circus aeruginosus*)<br>Griffon vulture (*Gyps fulvus*)<br>Black kite (*Milvus migrans*)	Buffers on the bone surface of fractures	**9 (90)**	**10**	*Enterococcus faecalis* (4)<br>*Pantoea agglomerans* (2)<br>*Proteus mirabilis* (1)<br>*Shigella flexneri* (1)<br>*Shigella boydii* (1)<br>*Leclercia adecarboxylata* (1)	Tardón et al. [36]
	2019-2020	Eurasian griffon vultures (Gyps fulvus)	cloacal swabs	**87 (39.9)**	**218**	*Escherichia coli* (87)	Guitart-Matas et al. [12]
Germany	2006/2008	Common Buzzard (*Buteo buteo*)<br>Eurasian Sparrowhawk (*Accipiter nisus*)	cloacal swabs/ postmortem	**62 (95.38)**	**65**	*Escherichia coli*	Guenther et al. [14]
	2010	Black Kites (*Milvus migrans*)<br>Red Kites (*Milvus milvus*)<br>Buzzards (*Buteo buteo*)<br>Sea Eagles (*Haliaeetus albicilla*)<br>Spotted Eagle (*Aquila pomarina*)<br>Goshawks (*Accicepter gentilis*)	cloacal swabs	**65 (38)**	**171**	*Escherichia coli*	Guenther et al. [37]
	2013	Black kite (*Milvus migrans*)	cloacal swabs	**1 (100)**	**1**	*Salmonella enterica* subsp. *enterica* serovar Corvallis (1)	Fischer et al. [38]
	2011-2014	Sea eagle (*Haliaeetus albicilla*)<br>Goshawk (*Accipiter gentilis*)<br>Sparrowhawk (*Accipiter nisus*)<br>Marsh harrier (*Circus aeruginosus*)<br>Buzzard (*Buteo buteo*)	cloacal swabs	**7**	**NA**	*E. coli* (7)	Schaufler et. al. [39]
Italy	2002-2010	Goshawk (*Accipiter gentilis*)<br>Sparrowhawk (*Accipiter nisus*)<br>Golden eagle (*Aquila chrysaetos*)<br>Common buzzard (*Buteo buteo*)<br>Short-toed snake-eagle (*Circaetus gallicus*)<br>Honey buzzard (*Pernis apivorus*)	cloacal swabs	**5**	**NA**	*Salmonella enterica* serotypes Typhimurium, Enteriditis, Ohio, Veneziana<br><br>*S. enterica subsp. arizonae*	Botti et al. [42]
	2012 –2014	Common buzzard (*Buteo buteo*)<br>Eurasian sparrowhawk (*Accipiter nisus*)<br>Short-toed eagle (*Circaetus gallicus*)	pellets	**NA**	**20**	NA	Dipineto et al. [40]

	2013	Booted eagle (*Hieraaetus pennatus*)<br>Common buzzard *(Buteo buteo)*<br>Pallid harrier *(Circus macrourus)*<br>European honey-buzzard (*Pernis apivorus*)	cloacal swabs	**NA**	**14**	NA	Giacopello et. al. [6]
	2016	Eurasian sparrowhawk (*Accipiter nisus*)<br>Common buzzard (*Buteo buteo*)<br>Eurasian marsh harrier (*Circus aeruginosus*)<br>European honey buzzard (*Pernis apivorus*)	postmortem	**5 (10.86)**	**46**	*E. coli* (4)<br>*Salmonella salamae* (1)	Gargiulo et al. [41]
UK	1995-2003	Sparrowhawk (*Accipiter nisus*)<br>Buzzard (*Buteo buteo*)	postmortem	**1 (9.09)**	**11**	*Salmonella* Typhimurium DT104	Pennycott et al. [43]
Netherlands	2010–2011	Common buzzard (*Buteo buteo*)<br>Northern goshawk (*Accipiter gentilis*)<br>Eurasian sparrowhawk (*Accipiter nisus*)<br>Western marsh harrier (*Circus aeruginosus*)	cloacal swabs/ postmortem	**0 (0)**	**18**	*Escherichia coli*	Veldman et al. [44]
Poland	2011-2013	Eurasian marsh harrier (*Circus aeruginosus*)<br>Sparrowhawk (*Accipiter nisus*)<br>Common buzzard (*Buteo buteo*)<br>Golden eagle (*Aquila chrysaetos*)	faeces	**1 (16.67)**	**6**	*Salmonella enterica* subsp. *enterica*	Krawiec et al. [45]
Austria	2013-2014	Bearded vulture (*Gypaetus barbatus*)<br>Common buzzard (*Buteo buteo*)<br>Golden eagle (*Aquila chrysaetos*)<br>Northern goshawk (*Accipiter gentilis*)<br>Harris's hawk (*Parabuteo unicinctus*)<br>European honey buzzard (*Pernis apivorus*)<br>Eurasian marsh harrier (*Circus aeruginosus*)<br>Pallid harrier *(Circus macrourus)*<br>White-tailed eagle (*Haliaeetus albicilla*)<br>Eurasian Sparrowhawk (*Accipiter nisus*)	cloacal swabs/ faeces/ postmortem	**NA**	**31**	*Salmonella* spp.<br>*Escherichia coli*	Konicek et. al. [46]
Czech Republic	2013-2014	Common buzzard (*Buteo buteo*)<br>Northern goshawk (*Accipiter gentilis*)<br>Eurasian marsh harrier (*Circus aeruginosus*)<br>Rough-legged buzzard (*Buteo lagopus*)<br>Black Kites (*Milvus migrans*)	cloacal swabs	**NA**	**47**	*Salmonella* spp.<br>*Escherichia coli*	Konicek et. al. [46]
Slovak Republic	2017	Golden eagle (*Aquila chrysaetos*)<br>Goshawks (*Accipiter gentilis*)	faeces	**19 (100)**	**19**	*Escherichia coli* (19)	Handrova and Kmet [7]
Switzerland	2018	Common buzzard (*Buteo buteo*)<br>Red kite (*Milvus milvus*)	cloacal swabs	**3**	**NA**	*Escherichia coli*	Zurfluh et al. [47]

			Total	**472 (33.81)**	**1396**		
Falconiformes							
Spain	3 years period	Kestrel *(Falco tinnunculus)*<br>Lesser kestrel *(Falco naumanni)*<br>Peregrine *(Falco peregrinus)* Hobby *(Falco subbteo)*<br>Merlin *(Falco columbarius)*	faeces/ cloacal swabs	**13 (4.34)**	**300**	*Salmonella* serotype Havana (7), Brandenburg (1), Adelaide (1), Hadar PT1 (1)	Reche et al. [27]
	2001-2002	Common kestrel (*Falco tinnunculus*)<br>Hobby (*Falco subbuteo*) Peregrine falcon (*Falco peregrinus*)	post-mortem	**1 (20)**	**5**	*Salmonella enterica* serotype 6,14:z4,z23	Millán et al. [21]
	2013-2014	Common kestrel (*Falco tinnununculus*)	cloacal swabs	**2 (12.5)**	**16**	*S. enterica* subsp. *enterica* serovar: Typhimurium (1), Enteritidis (1)	Molina-López et al. [31]
	2013-2014	Merlin (*Falco columbarius*)<br>Common kestrel (*Falco tinnunculus*)	cloacal swabs	**0 (0)**	**8**	*E. coli*	Alcalá et. al. [29]
	2012-2014	Lesser kestrel (*Falco naumanni*)<br>Common kestrel (*Falco tinnunculus*)<br>Peregrine falcon (*Falco peregrinus*)	faeces	**5 (5.88)**	**85**	*Salmonella* Typhimurium (2), Enteritidis (1), London(1). *Salmonella* spp. (1)	Jurado-Tarifa et. al. [30]
	2015-2016	Lesser kestrel (*Falco naumanni*)<br>Common kestrel (*Falco tinnunculus*)<br>Peregrine falcon (*Falco peregrinus*)	cloacal swabs	**6**	**NA**	*Escherichia coli* (1), *Klebsiella pneumoniae* (2), *Hafnia alvei* (1), *Enterobacter* spp. (2)	Oteo et al. [35]
	2019	Common kestrel (*Falco tinnunculus*)	buffers on the bone surface of fractures	**1 (33.33)**	**3**	*Hafnia alvei*	Tardón et al. [36]
Italy	2002-2010	Peregrine falcon (*Falco peregrinus*)<br>Hawk (*Falco* spp.)<br>Common kestrel (*Falco tinnunculus*)	cloacal swabs	**2**	**NA**	*Salmonella enterica* serotype Brancaster, Veneziana	Botti et al. [42]
	2012 –2014	Common kestrel (*Falco tinnunculus*)<br>Peregrine falcon (*Falco peregrinus*)	pellets	**NA**	**40**	*NA*	Dipineto et al. [40
	2013	Common kestrel *(Falco tinnunculus)*<br>Peregrine harrier *(Falco peregrinus)*<br>Eleonora's falcon *(Falco eleonorae)*	cloacal swabs	**NA**	**14**	NA	Giacopello et. al. [16]
	2016	Saker falcon (*Falco cherrug*)<br>Peregrine falcon (*Falco peregrinus*)<br>Eurasian hobby (*Falco subbuteo*)<br>Common kestrel (*Falco tinnunculus*)	post-mortem	**10 (17)**	**59**	*E. coli* (3)<br>*Salmonella salamae* (2),<br>*Salmonella* Napoli (4),<br>*Salmonella* Typhimurium (1)	Gargiulo et al. [41]
Germany	2006/2008	Common Kestrel (*Falco tinnunculus*)	cloacal swabs	**6 (50)**	**12**	*Escherichia coli* (6)	Guenther et al. [14]
Netherlands	2010–2011	Common kestrel (*Falco tinnunculus*)	cloacal swabs/ postmortem	**0 (0)**	**1**	*Escherichia coli*	Veldman et al. [44]
Austria	2013-2014	Eurasian hobby (*Falco subbuteo*)<br>Common kestrel (*Falco tinnunculus*)	cloacal swabs/ faeces/ post-mortem	**NA**	**68**	*Salmonella* spp.<br>*Escherichia coli*	Konicek et. al. [46]

Czech Republic	2013-2014	Common kestrel (*Falco tinnunculus*)	cloacal swabs	**NA**	**15**	*Salmonella* spp.<br>*Escherichia coli*	Konicek et. al. [46]
Switzerland	2018	Eurasian hobby (*Falco subbuteo*)	cloacal swabs	**1**	**NA**	*Escherichia coli*	Zurfluh et al. [47]
			Total	**47 (7.5)**	**626**		
			Strigiformes				
Norway	1980-1981	Tengmalm's owl (*Aegolius funereus*)	cloacal swabs	**2 (16.7)**	**12**	*Yersinia* spp. (2)	Kapperud & Rosef [48]
	1969–2000	Tengmalm's Owl (*Aegolius funereus*)	post-mortem	**1**	**NA**	*Salmonella enterica* serovar Typhimurium O:4,12	Refsum et al. [17]
Spain	3 years period	Long-eared owl (*Asio otus*)<br>Scops owl (*Otus scops*)<br>Little owl (*Athene noctua*)<br>Short-eared owl (*Asio flammeus*<br>Eagle owl (*Bubo bubo*)<br>Tawny owl (*Strix aluco*<br>Barn owl (*Tyto alba*)	faeces / cloacal swabs	**12 (10.1)**	**119**	*Salmonella* serotype Havana (4),<br>Newport (2),<br>Enteritidis PT6a (1),<br>Enteritidis PNR (1),<br>Adelaide (1),<br>Hadar PT5 S84 (1),<br>Saintpaul S86 (1),<br>Typhimurium DT104 (1)	Reche et al. [27]
	2001-2002	Tawny owls (*Strix aluco*)<br>Barn owl (*Tyto alba*)<br>Long-eared owl (*Asio otus*)<br>Eagle owl (*Bubo bubo*)<br>Little owl (*Athene noctua*)	post-mortem	**1 (20)**	**5**	*Salmonella enterica* serotype Enteritidis	Millán et al. [21]
	2012-2014	Long-eared owl (*Asio otus*)<br>Eagle owl (*Bubo bubo*)<br>Tawny owl (*Strix aluco*)<br>Short-eared owl (*Asio flammeus*)<br>Barn owl (*Tyto alba*)<br>Little owl (*Athene noctua*)<br>European scops owl (*Otus scops*)	faeces	**9 (4.69)**	**192**	*Salmonella* Typhimurium (2),<br>Enteritidis (2), Mikawasima (1)<br>*Salmonella* spp. (4)	Jurado-Tarifa et. al. [30]
	2013-2014	Eurasian eagle-owl (*Bubo bubo*)<br>Barn owl (*Tyto alba*)<br>Tawny owl (*Strix aluco*)	cloacal swabs	**3 (12)**	**25**	*S.* Typhimurium monophasic 4,12:i:- (2)<br>*S. Hadar (1)*	Molina-López et al. [31]
	2013 -2014	Barn owl (*Tyto alba*)<br>Eurasian Eagle-owl (*Bubo bubo*)<br>Long eared owl (*Asio otus*), Eurasian scops owl (*Otus scops*)	cloacal swabs	**1 (5.9)**	**17**	*Escherichia coli*	Alcalá et. al. [29]
	2015 - 2016	Eurasian eagle-owl (*Bubo bubo*)<br>Barn owl (*Tyto alba*)<br>Tawny owl (*Strix aluco*)	cloacal swabs	**8**	**NA**	*Escherichia coli* (4)<br>*Klebsiella pneumoniae* (4)	Oteo et al. [35]
	2019	Eurasian Eagle-owl (*Bubo bubo*)	buffers on the bone surface of fractures	**4 (80)**	**5**	*Escherichia fergusonii (4)*<br>*Enterococcus faecalis (2)*<br>*Hafnia alvei (1)*<br>*Shigella flexneri (1)*<br>*Shigella sonnei (1)*	Tardón et al. [36]

UK	1995-2003	Tawny owl (*Strix aluco*)	postmortem	**2 (50)**	**4**	*Salmonella enterica* serovar Typhimurium DT41	Pennycott et al. [43]
Germany	2006-2008	Barn Owl (*Tyto alba*)<br>Long-eared Owl (*Asio otus*)	cloacal swabs/ post-mortem	**10 (100)**	**10**	*Escherichia coli*	Guenther et al. [14]
Italy	2002-2010	Long-eared owl (*Asio otus*)<br>Scops owl (*Otus scops*)<br>Tawny owl (*Strix aluco*)<br>Barn owl (*Tyto alba*)	cloacal swabs	**6**	**NA**	*Salmonella enterica* subspecies *enterica*<br>Serotypes: Livingstone (1)/ Loanda (1)/ Suberu (1)/ *S*. Typhimurium (2), *S*. Enteritidis (1)	Botti et al. [42]
	2012 -2014	Barn owl (*Tyto alba*)<br>Tawny owl (*Strix aluco*)<br>Eurasian eagle-owl (*Bubo bubo*)<br>Short-eared owl (*Asio flammeus*)	pellets	**NA**	**13**	NA	Dipineto et al. [40]
	2015-2016	Long-eared owl (*Asio otus*)<br>Little owl (*Athene noctua*)<br>Eurasian scops owl (*Otus scops*)<br>Tawny owl (*Strix aluco*)<br>Barn owl (*Tyto alba*)	post-mortem	**5 (11.62)**	**43**	*E. coli* O26 (1), O145 (2)<br>*Salmonella* Typhimurium (2)	Gargiulo et al. [41]
Austria	2013-2014	Long-eared owl (*Asio otus*)<br>Little owl (*Athene noctua*)<br>Eurasian eagle-owl (*Bubo bubo*)<br>Tawny owl (*Strix aluco*)<br>Barn owl (*Tyto alba*)	cloacal swabs/ faeces/ post-mortem	**NA**	**32**	*Salmonella* spp.<br>*Escherichia coli*	Konicek et. al. [46]
Czech Republic	2013-2014	Long-eared owl (*Asio otus*)<br>Tawny owl (*Strix aluco*)	cloacal swabs	**NA**	**10**	*Salmonella* spp.<br>*Escherichia coli*	Konicek et. al. [46]
			Total	**64 (13.2)**	**486**		
			Columbiformes				
Norway	1980-1981	Domestic pigeon (*Columba livia*)	cloacal swabs	**0 (0)**	**71**	*Yersinia* spp.<br>*Salmonella* spp.	Kapperud & Rosef [48]
	1969–2000	Rock dove (*Columba livia*)	post-mortem	**3 (4.17)**	**72**	*Salmonella* Typhimurium O:4,12 (1), O:4,5,12 (2)	Refsum et al. [17]
	2003	Feral pigeon (*Columba palumbus*)	faeces/ cloacal swabs/ post-mortem	**0 (0)**	**200**	*Salmonella* spp.	Lillehaug et al. [49]
Czech Republic	1984-1991	Feral pigeon (*Columba livia f. domestica*)	cloacal swabs/ post-mortem	**2 (0.46)**	**432**	*Salmonella typhimurium* var. Copenhagen biotype 27 hi (2)	Cízek et. al. [50]
	2013-2014	Feral pigeon (*Columba livia f. domestica*)<br>Common wood pigeon (*Columba palumbus*)	faeces/ cloacal swabs	**281 (92.1)**	**305**	*Salmonella* spp. (0)<br>*Escherichia coli* (281)	Konicek et. al. [46]
Italy	1997-1998	Feral pigeon (*Columba livia*)	faeces	**70 (10.7)**	**649**	*Escherichia coli*	Morabito et al. [51]

	2002-2010	Common pigeon (*Columba livia*)	cloacal swabs	**4**	**NA**	*Salmonella enterica* subspecies *enterica* serotypes Farsta (1), Tsevie (1), Typhimurium (2)	Botti et al. [42]
	2010-2013	Pigeons (*Columbia livia*)<br>Eurasian collared dove (*Streptopelia dedecaocto*)	postmortem	**0 (0)**	**5**	*Salmonella* spp.	Rubini et. al. [52]
Finland	1998	Pigeon (*Columba palumbus*)	cloacal swabs	**2 (7)**	**29**	*Escherichia coli* O45	Kobayashi et al. [16]
UK	1995-2003	Feral pigeon (*Columba livia*)<br>Wood pigeon (*Columba palumbus*)<br>Collared dove (*Streptopelia decaocto*)	postmortem	**5 (5.75)**	**87**	*Salmonella enterica* serovar Typhimurium DT2 (2), DT99 (2)<br>*Salmonella* Liverpool (1)	Pennycott et al. [43]
	2006	Collared dove (*Streptopelia decaocto*)<br>Wood pigeon (*Columba palumbus*)	postmortem	**2**	**NA**	*Salmonella enterica* ser. Typhimurium DT56 4,12:i (2)	Hughes et al. [53]; Hughes et al. [54]
	2016	Pigeon (*Columba livia*)<br>Wood Pigeon (*Columba palumbus*)	faeces/ postmortem	**NA**	**20**	*Escherichia coli* (27)	Swift et al. [11]
Denmark	2001-2002	Collared Dove (*Streptopelia decaocto*)<br>Domestic/feral pigeon (*Columba livia f. domestica*)	cloacal swabs	**0 (0)**	**11**	*Salmonella* spp.	Skov et al. [26]
Germany	2006/2008	Eurasian Collared Dove (*Streptopelia decaocto*)<br>Rock Pigeon (*Columba livia*)	cloacal swabs	**20 (100)**	**20**	*Escherichia coli*	Guenther et al. [14]
	2011-2014	Pigeon (*Columba livia*)	cloacal swabs	**1**	**NA**	*Escherichia coli*	Schaufler et. al. [39]
Spain	2009-2011	Rock Pigeon (*Columba livia*)	faeces	**2**	**NA**	*Salmonella enterica* DT164 (2)	Andrés et al. [55]
	2013-2014	Common wood pigeon (*Columba palumbus*)<br>Eurasian collared dove (*Streptopelia decaocto*)	cloacal swabs	**0 (0)**	**5**	*Escherichia coli*	Alcalá et. al. [29]
	2018 - 2019	Common wood pigeon (*Columba palumbus*)	cloacal swabs	**7 (7)**	**100**	*Salmonella* Typhimurium (4)<br>*S.* Enteritidis (3)	Martin-Maldonado et al. [56]
Netherlands	2010–2011	Feral pigeon (*Columba livia domesticus*)<br>Common wood pigeon (*Columba palumbus*)<br>Stock dove (*Columba oenas*)	postmortem	**1 (14.3)**	**7**	*Escherichia coli*	Veldman et al. [44]
Switzerland	2012	Feral pigeon (*Columba livia f. domestica*)	postmortem/ cloacal swabs	**3 (1)**	**298**	*Escherichia coli* (4)	Zurfluh et al. [57]
	2018	Feral pigeon (*Columba livia domestica*)<br>Eurasian collared dove (*Streptopelia decaocto*)	cloacal swabs	**4**	**NA**	*Escherichia coli (4)*	Zurfluh et al. [47]

Austria	2013-2014	Feral pigeon (*Columba livia f. domestica*)<br>Eurasian collared dove (*Streptopelia decaocto*)<br>Homing pigeon (*Columba livia domestica*)<br>Common wood pigeon (*Columba palumbus*)	postmortem/ cloacal swabs/ faeces	**165 (63.7)**	**259**	*Salmonella* Typhimurium *(1)*<br>*Escherichia coli (164)*	Konicek et. al. [46]
Poland	2011-2013	Common wood pigeon (*Columba palumbus*)	faeces/ postmortem	**1 (16.67)**	**6**	*Salmonella enterica* subspecies *enterica*<br>serotype Typhimurium 4,12:i:1,2	Krawiec et al. [45]; Krawiec et al. [58]
France (Reunion Island)	2011-2013	Pigeon (*Columba livia*)	cloacal swabs/ postmortem	**0 (0)**	**30**	*Salmonella* spp.	Tessier et. al. [59]
France	2016	Pigeons (*Columba livia* f. urbana)	faeces	**3 (4.22)**	**71**	*Escherichia coli* (1)<br>*Hafnia alvei* (1)<br>*Panteoa ananatis* (1)	Ngaiganam et al. [60]
			Total	**576 (21.51)**	**2677**		
			Corvidae				
Norway	1969–2000	Black-billed Magpie (*Pica pica*)<br>Hooded Crow (*Corvus corone*)	postmortem	**2 (2.17)**	**92**	*Salmonella enterica* serovar Typhimurium O:4,5,12 (1);<br>*S. enterica* serovar Paratyphi B var. Java (1)	Refsum et al. [17]
	1979	Carrion crows (*Corvus corone*)	rectal swabs	**2 (50)**	**4**	*Yersinia enterolitica (3)*	Kapperud [61]
	1980-1981	Raven (*Corvus corax*)<br>Hooded crow (*Corvus corone cornix*)	cloacal swabs	**0 (0)**	**50**	*Yersinia* spp.<br>*Salmonella* spp.	Kapperud & Rosef [48]
UK	1995-2003	Rook (*Corvus frugilegus*)<br>Carrion crow (*Corvus corone corone*)<br>Jackdaw (*Corvus monedula*)<br>Magpie (*Pica pica*)	postmortem	**2 (3.63)**	**55**	*Salmonella enterica* serovar Typhimurium DT40, DT41	Pennycott et al. [43]
Spain	2001-2002	Magpie (*Pica pica*)<br>Carrion crow (*Corvus corone*)	postmortem	**0 (0)**	**7**	*Salmonella enterica*	Millán et al. [21]
	2011	Corn crows/ Rooks (*Corvus frugilegus*)	faeces	**2 (1.34)**	**150**	*Salmonella* Oranienburg (2)	Janecko et al. [62]
	2015-2016	Common raven (*Corvus corax*)	cloacal swabs	**1**	**NA**	*Escherichia coli*	Oteo et al. [35]
Denmark	2001-2002	Rook (*Corvus frugilegus*)	cloacal swabs	**0 (0)**	**2**	*Salmonella* spp.	Skov et al. [26]
Italy	2002-2010	Eurasian jay *(Garrulus glandarius)*<br>Carrion crow (*Corvus corone cornix*)<br>Magpie (*Pica pica*)	cloacal swabs	**2**	**NA**	*Salmonella* Typhimurium (1)<br>*Salmonella enterica* subspecies *enterica* serotype Infantis (1)	Botti et al. [42]
	2011	Rooks (*Corvus frugilegus*)	faeces	**0 (0)**	**150**	*Salmonella* spp.	Janecko et al. [62]
	2013	Eurasian jackdaw (*Corvus monedula*)<br>Carrion crow (*Corvus corone cornix*)<br>Eurasian jay *(Garrulus glandarius)*	cloacal swabs	**NA**	**4**	NA	Giacopello et. al. [6]
	2010 - 2013	Magpie (*Pica pica*)<br>Hooded crow (*Corvus corone cornix*)<br>Jay (*Garrulus glandarius*)	postmortem	**9 (1.08)**	**831**	*S.* Typhimurium (5), Bredeney (1), Braenderup (1), Enteritidis (1), Mbandaka (1)	Rubini et. al. [52]

Germany	2006-2008	Eurasian Jay (*Garrulus glandarius*)<br>Jackdaw (*Corvus monedula*)<br>Rook (*C. frugilegus frugilegus*)	cloacal swabs	**18 (81.81)**	**22**	*Escherichia coli* (18)	Guenther et al. [14]
	2011	Rooks (*Corvus frugilegus*)	faeces	**1 (1)**	**100**	*Salmonella* Senftenberg (1)	Janecko et al. [62]
	2011-2014	Crow (*Corvus corone*)<br>Magpie (*Pica pica*)	cloacal swabs	**3**	**NA**	*E. coli* (3)	Schaufler et. al. [39]
Netherlands	2010–2011	Black crows (*Corvus corone*)	postmortem	**0 (0)**	**1**	*Escherichia coli*	Veldman et al. [44]
Czech Republic	2010-2013	Rooks (*Corvus frugilegus*)<br>Common ravens (*Corvus corax*)	faeces	**4 (0.76)**	**525**	*Salmonella* Typhimurium (2)<br>S. Hadar (1)<br>S. Oranienburg (1)	Janecko et al. [62]
	2013-2014	Carrion crow (*Corvus corone*)<br>Eurasian jackdaw (*Corvus monedula*)<br>Eurasian jay *(Garrulus glandarius)*<br>Rook (*Corvus frugilegus*)<br>Eurasian magpie (*Pica pica*)	cloacal swabs	**NA**	**51**	*Salmonella* spp.<br>*Escherichia coli*	Konicek et. al. [46]
France	2011	Rooks (*Corvus frugilegus*)	faeces	**3 (9.68)**	**31**	*Salmonella* Montevideo (3)	Janecko et al. [62]
Serbia	2011	Rooks (*Corvus frugilegus*)	faeces	**0 (0)**	**150**	*Salmonella* spp.	
Poland	2011	Rooks (*Corvus frugilegus*)	faeces	**2 (0.67)**	**298**	*Salmonella* Enteritidis (2)	
	2011-2013	Rook (*Corvus frugilegus*)<br>Hooded crow (Corvus cornix)	faeces/ postmortem	**1 (8.33)**	**12**	*Salmonella enterica* subspecies *enterica*	Krawiec et al. [45]
	2022-2023	Rook (*Corvus frugilegus*)	faeces	**31 (52)**	**60**	*Escherichia coli*	Łopucki et al. [63]
Switzerland	2011	Rooks (*Corvus frugilegus*)	faeces	**0 (0)**	**49**	*Salmonella* spp.	Janecko et al. [62]
	2018	Carrion crow (*Corvus corone*)	cloacal swabs	**2**	**NA**	*Escherichia coli*	Zurfluh et al. [47]
Slovakia	2013	Common ravens (*Corvus corax*)	faeces	**9 (3.15)**	**286**	*Salmonella* Infantis (4)<br>*S.* Enteritidis (3)<br>*S.* Senftenberg (2)	Janecko et al. [62]
Austria	2013-2014	Carrion crow (*Corvus corone*)<br>Eurasian jackdaw (*Corvus monedula*)<br>Eurasian jay *(Garrulus glandarius)*<br>Rook (*Corvus frugilegus*)<br>Eurasian magpie (*Pica pica*)	cloacal swabs/ faeces/ post-mortem	**NA**	**130**	*Salmonella* spp.<br>*Escherichia coli*	Konicek et. al. [46]
			Total	**94 (3)**	**3060**		
			Laridae				
Norway	1980-1981	Herring gull (*Larus argentatus*)<br>Black-headed gull (*Larus ridibundus*)<br>Common gull (*Larus canus*)	cloacal swabs	**6 (2.8)**	**216**	*Yersinia* spp. (2)<br>*Salmonella* spp. (4):<br>*S. typhimurium* (2),<br>*S. indiana* (1), and	Kapperud & Rosef [48]

		Lesser black-backed gull (*Larus fuscus*)<br>Great black-backed gull (*Larus marinus*)<br>Common tern (*Sterna hirundo*)				*S. djugu* (1)	
	1969–2000	Black-headed gull (*Larus ridibundus*)<br>Herring gull (*Larus argentatus*)<br>Mew gull (*Larus canus*)	Post-mortem	**15**	**N.A.**	*Salmonella enterica* serovar Typhimurium O:4,12 (5) and O:4,5,12 (10)	Refsum et al. [17]
	2000-2001	Great black-backed gull (*Larus marinus*)<br>Herring gull (*Larus argenatus*)	cloacal swabs/ post-mortem	**31 (7.34)**	**422**	*Salmonella* Agona (4), Montevideo (14), Senftenberg (13)	Nesse et al. [64]
	2010	Glaucous gull (*Larus hyperboreus*)	faeces	**2 (13.33)**	**15**	*Enterobacter cloacae (2)*	Literak et al. [65]
UK	1995-2003	Black-headed gull (*Larus ridibundus*)<br>Common gull (*Larus canus*)<br>Great black-backed gull (*Larus marinus*)<br>Immature herring gull (*Larus argentatus*) or lesser black-backed gull (*Larus fuscus*)	postmortem	**7 (7.21)**	**97**	*S.* Typhimurium DT41 (5) and DT195 (2)	Pennycott et al. [43]
	2005	Black-headed gull (*Larus ridibundus*),<br>Herring gull (*Larus argentatus*)	postmortem	**4**	**NA**	*Salmonella* Typhimurium (1),<br>*S.* Newport (1),<br>*S.* Senftenberg (1),<br>1 unclassified *Salmonella* isolate	Hughes et al. [53]; Hughes et al. [54]
	2015	Gulls (Larus spp.)	faeces	**NA**	**NA**	*Salmonella enterica* serovar 4,5,12:i:- (monophasic S. Typhimurium)<br>*Salmonella* Rissen	De Lucia et al. [66]
Switzerland	2018	Yellow-legged gulls (*Larus michahellis*)	cloacal swabs	**1**	**NA**	*Escherichia coli*	Zurfluh et al. [47]
Czech Republic	1984-1991	Black-headed gull (*Larus ridibundus*)<br>Common gull (*Larus canus*)	cloacal swabs/ postmortem	**151 (16.24)**	**930**	*S. typhimurium*<br>*S. enteriditis*<br>*S. agona*<br>*S. isangi*<br>*S. thompson*<br>*S. hadar*<br>*S. schwarzengrund*<br>*S. panama*<br>*S. derby*<br>*S. berta*<br>*S. montevideo*<br>*S. infantis*<br>*S. abony*	Cízek et al. [50]
	1992-1993	Black-headed gull (*Larus ridibundus*)	cloacal swabs	**38 (24.7)**	**154**	*S. typhimurium* (31)<br>*S. enteritidis* (5)<br>*S. panama* (1)<br>*S. anatum* (1)	Hubálek et al. [67]

	2018-2019	Caspian gull (*Larus cachinnans*)	cloacal swabs	**79 (67.5)**	**117**	*Escherichia coli* (141), *Escherichia albertii* (17)	Nesporova et al. [68]
Sweden	1997	Black-headed gull (*Larus ridibundus*) Common gull (*L. canus*)	faeces	**2 (4)**	**50**	*S. typhimurium* (2)	Palmgren et al. [69]
	1998-1999	Herring gull (*Larus argentatus*)	pooled samples	**4 (3.6)**	**111**	*Salmonella* Oranienburg (1), *S.* Livingstone (1), *S.* Agona (1), *S.* Typhimurium DT 195 (1)	Wahlström et al. [70]
	2008	Black-headed gull (*Larus ridibundus*)	cloacal swabs	**83 (83)**	**100**	*Escherichia coli*	Bonnedahl et al. [71]
	2013	Great black-backed gull (*Larus marinus*), Herring gull (*Larus argentatus*), Common gull (*Larus canus*), Black-headed gull (*Croicocephalus ridibundus*)	faeces	**29 (17)**	**170**	*Escherichia coli* (29)	Atterby et al. [23]
Finland	1998	Black-headed gull (*Larus ridibundus*), Black-headed gull (*L. argentatus*)	cloacal swabs	**34 (40)**	**86**	*Escherichia coli*	Kobayashi et al. [16]
Denmark	2001-2002	Common gull (*Larus canus*)	cloacal swabs	**0 (0)**	**2**	*Salmonella* spp.	Skov et al. [26]
France	2009	Yellow-legged Gulls (*Larus michahellis*)	cloacal swabs	**153 (85)**	**180**	*Escherichia coli*	Bonnedahl et al. [13]
	2016	Yellow-legged gulls (*Larus michahellis*)	faeces	**9 (24.32)**	**37**	*Escherichia coli* (3) *Cronobacter sakazakii* (1) *Hafnia alvei* (5) *Proteus hauseri* (1) *Serratia marcescens* (1)	Ngaiganam et al. [60]
Germany	2006-2008	Black-headed gull (*Chroicocephalus ridibundus*)	cloacal swabs/ postmortem	**1 (50)**	**2**	*Escherichia coli*	Guenther et al. [14]
	2011-2014	Herring gull (*Larus argentatus*)	cloacal swabs	**1**	**NA**	*Escherichia coli*	Schaufler et. al. [39]
Poland	2008-2009	Herring gull (*Larus argentatus*)	cloacal swabs	**18 (66.7)**	**27**	*Escherichia coli*	Literak et al. [72]
Italy	2002-2010	Seagull (*Larus* spp.)	cloacal swabs	**1**	**NA**	*S.* Enteritidis	Botti et al. [42]
Netherlands	2010–2011	European herring gull (*Larus argentatus*), Black-headed gull (*Chroicocephalus ridibundus*), Lesser black-backed gull (*Larus fuscus*), Great black-backed gull (*Larus marinus*), and common gull (*Larus canus*) Common tern (*Sterna hirundo*)	cloacal swabs /post-mortem	**29 (18.83)**	**154**	*Escherichia coli*	Veldman et al. [44]

Ireland	2013	Herring gull (*Larus argentatus*)<br>Black-headed gull (*Larus ridibundus*)<br>Lesser black-backed gull (*Larus fuscus*)	faeces	**70 (77.8)**	**90**	*Escherichia coli*	Carroll et al. [73]
	2019-2020	Lesser black-backed gull (*Larus fuscus*),<br>Black-headed gull (*Chroicocephalus ridibundus*),<br>European herring gull (*Larus argentatus*)	faeces	**NA**	**28**	*Escherichia coli*	Martin et al. [83]
Spain	2013 - 2014	Yellow-legged gull (*Larus michahellis*)	cloacal swabs	**1 (100)**	**1**	*Escherichia coli*	Alcalá et. al. [29]
	2015 - 2016	Black-headed gull (*Croicocephalus ridibundus*)<br>Lesser black-backed gull (*Larus fuscus*)	cloacal swabs	**15**	**NA**	*Escherichia coli* (14)<br>*Proteus mirabilis* (1)	Oteo et al. [35]
	2018-2019	Lesser black-backed gull (*Larus fuscus*)	cloacal swabs	**5 (20)**	**25**	*Salmonella* spp.:<br>*S.* Typhimurium (1)<br>*S.* Typhimurium monophasic variant (mST) (1)<br>*S.* Infantis (1)<br>*S.* Kentucky (1)<br>*S.* Saintpaul (1)	Martin-Maldonado et al. [56]
	2013, 2009-2010, 2018	Audouin's gull (*Larus audouinii*)<br>Yellow-legged gull (*Larus michahellis*)	cloacal swabs	**45 (10.5)**	**429**	*Salmonella enterica* (various serovars):<br>*S.* Typhimurium,<br>*S.* Typhimurium monophasic variant,<br>*S.* Bredeney (4),<br>*S.* Chester (2), *S.* Goldcoast (3),<br>*S.* Sandiego (3), *S.* Wangata (2) and serovars Corvallis (3), Infantis (2), London (1), Mons (1), Virchow (1)	Manzanares-Pedrosa et al. [74]
Spain and Portugal	2009	Yellow-legged gulls (*Larus michahellis*)	faeces	**NA**	**NA**	*Escherichia coli* (5), *Klebsiella pneumoniae* (1)	Ahlstrom et al. [18]
			Total	**836 (24.3)**	**3443**		
			**Aquatic birds**				
			Anseriformes				
UK	1969-1970	Tufted duck (*Aythya fuligula*)<br>Pochard (*Aythya ferina*)<br>Teal (*Anas crecca*)<br>Widgeon (*Anas penelope*)	faeces	**20 (4.2)**	**477**	*S. typhimurium* (18)<br>*S. paratyphi* B (1)<br>*S. emek* (1)	Mitchell & Ridgwell [75]

		Gadwall (*Anas strepera*)					
	1995-2003	Mute swan (*Cygnus olor*)	postmortem	**0 (0)**	**23**	*Salmonella* spp.	Pennycott et al. [43]
Norway	1980-1981	Goldeneye (*Bucephala clangula*)	cloacal swabs	**0 (0)**	**1**	*Yersinia* spp.<br>*Salmonella* spp.	Kapperud & Rosef [48]
	1969-2000	Mallard (*Anas platyrhynchos*)<br>Canada Goose (*Branta canadensis*)	postmortem	**4**	**NA**	*Salmonella enterica* serovar Typhimurium O:4,5,12 (4)	Refsum et al. [17]
Sweden	1998-1999	Canada geese (*Branta canadensis*)	faeces/ postmortem	**0 (0)**	**105**	*Salmonella* spp.	Wahlström et al. [70]
	2013	Urban wild mallard (*Anas platyrhynchos*)	faeces	**386 (47)**	**813**	*Escherichia coli* (386),<br>*Klebsiella pneumoniae* (25)	Hessman et al. [76]
Spain	2008-2011	Mallard (*Anas platyrhynchos*)<br>Eurasian teal (*Anas crecca*)<br>Northern shoveler (*Anas clypeata*)<br>Eurasian wigeon (*Anas penelope*)<br>Common pochard (*Aythya ferina*)<br>Northern pintail (*Anas acuta*)<br>Red-crested Pochard (*Netta rufina*)<br>Gadwall (*Anas strepera*)	cloacal swabs	**0 (0)**	**277**	*Salmonella* spp.	Antilles et al. [77]
	2013-2014	Mallard (*Anas platyrhynchos*)	cloacal swabs	**0 (0)**	**1**	*Escherichia coli*	Alcalá et. al. [29]
Poland	2008-2009	Mallard (*Anas platyrhynchos*)	cloacal swabs	**65 (75.6)**	**86**	*E. coli*	Literak et al. [72]
	2011-2013	Mallard duck (*Anas platyrhynchos*)<br>Mute swan (*Cygnus olor*)<br>Whooper swan (*Cygnus cygnus*)	cloacal swabs/ faeces	**8 (5.2)**	**154**	*Salmonella enterica* enterica Typhimurium 4,12:i:1,2 (3), Hadar 6,8:z10:e,n,x (1)<br>*Salmonella enterica (4)*<br>*Salmonella enterica* subsp. *houtenae (IV) (1)*	Krawiec et al. [45]
	2011-2013	Mallard (*Anas platyrhynchos)*<br>Mute swan (*Cygnus olor)*	cloacal swabs	**75 (100)**	**75**	*Escherichia coli* (75)	Kuczkowski et. al. [78]
	2011-2014	Mallard duck (*Anas platyrhynchos*)	NA	**5**	**NA**	*Salmonella enterica* subsp. *enterica* (1), Typhimurium (3), *houtenae* (1)	Krawiec et al. [58]
	2024	Mallard (*Anas platyrhynchos*)	postmortem	**1 (100)**	**1**	*Salmonella enterica* subsp. *diarizonae* 58:r:z53 (1)	Wodz et al. [79]
Belgium	2011	Canada Geese (*Branta canadensis*)<br>Greylag swan (*Anser anser domesticus*)	cloacal swabs	**2 (0.5)**	**396**	*Escherichia coli* (2)	Garmyn et. al. [80]
Italy	1991	Northern shoveler (*Anas clypeata*)	intestinal content	**1 (100)**	**1**	*Yersinia mollaretii*	Iannibelli et al. [81]
	2010-2013	Whooper swan (*Cygnus cygnus*)<br>Wild duck (*Anas platyrhynchos*)	postmortem	**0 (0)**	**2**	*Salmonella* spp.	Rubini et. al. [52]
Netherlands	2010-2011	Duck (*Anas platyrhynchos*)<br>Geese (*Anser anser*)	cloacal swabs/ postmortem	**21 (18.26)**	**115**	*Escherichia coli* (21)	Veldman et al. [44]

		Swan (Anatidae/Cygnini)				
	2011-2013	Greylag goose (*Anser anser)*<br>Canada goose (*Branta canadensis)*	cloacal swabs	**94 (100)**	**94**	*Escherichia coli* (94)	Kuczkowski et. al. [78]
Germany	2007-2011	Mallard ducks (*Anas platyrhynchos*)	faeces	**400**	**NA**	*Escherichia coli* (400)	Rödiger et al. [82]
	2011-2014	Mute swan (*Cygnus olor*)<br>Bean goose (*Anser fabalis*)	cloacal swabs	**11**	**NA**	*Escherichia coli*	Schaufler et. al. [39]
Austria	2013-2014	Canada goose (*Branta canadensis)*<br>Domestic goose (*A. anser × A. cygnoides)*<br>Mallard (*Anas platyrhynchos*)<br>Mandarin duck (*Aix galericulata*)<br>Muscovy duck (*Cairina moschata*)<br>Mute swan (*Cygnus olor*)	cloacal swabs/ faeces/ postmortem	**40 (80)**	**50**	*Salmonella* spp. (0)<br>*Escherichia coli* (40)	Konicek et. al. [46]
Czech Republic	1992-1993	Greylag goose (*Anser anser)*	cloacal swabs	**1 (4.76)**	**21**	*S. enteritidis*	Hubálek et al. [67]
	2013-2014	Mallard (*Anas platyrhynchos*)<br>Mute swan (*Cygnus olor*)<br>Greylag goose (*Anser anser)*	cloacal swabs	**50 (98)**	**51**	*Salmonella* Infantis (1)<br>*Salmonella* Enteritidis (1)<br>*Escherichia coli* (48)	Konicek et. al. [46]
Ireland	2013-2021	Brent goose (*Branta bernicla*)	faeces	**NA**	**16**	*Escherichia coli*	Martin et al. [83]
Ukraine	2020-2021	Eurasian wigeon (*Anas penelope*)<br>Eurasian teal (*Anas crecca*)<br>White-fronted goose (*Anser albifrons*)<br>Red-breasted goose (*Rufibrenta ruficollis*)<br>Graylag goose (*Anser anser*)<br>Shelduck (*Tadorna tadorna*)	cloacal swabs/ faeces	**19 (17)**	**111**	*Escherichia coli (19)*	Eckenko et al. [84]
			Total	**1203 (42)**	**2870**		
			Pelecaniformes				
Spain	2001-2002	Little egret (*Egretta garzetta)*<br>Grey heron (*Ardea cinerea)*	postmortem	**0 (0)**	**6**	*Salmonella* spp.	Millán et al. [21]
	2013-2014	Little bittern (*Ixobrychus minutus*)<br>Grey heron (*Ardea cinerea*)	cloacal swabs	**0 (0)**	**2**	*Escherichia coli*	Alcalá et. al. [29]
	2019	Cattle egret (*Bulbucus ibis*)<br>Glossy ibis (*Plegadis falcinellus*)	Buffers on the bone surface of fractures	**7**	**2**	*Enterobacter cloacae (1)*<br>*Enterobacter ludwigii (1)*<br>*Escherichia fergusonii (2)*<br>*Pantoea agglomerans (1)*<br>*Shigella flexneri (2)*	Tardón et al. [36]

Italy	2002-2010	Black-crowned night heron (*Nycticorax nycticorax*)	cloacal swab	**0**	**NA**	*Salmonella enterica*	Botti et al. [42]
	2012	Night herons (*Nycticorax nycticorax*)	faeces/ cloacal swab	**1 (7.7)**	**13**	*Salmonella* Typhimurium	Mancini et. al. [85]
	2013	Little egret (*Egretta garzetta)*<br>Grey heron (*Ardea cinerea*)<br>Black-crowned night heron (*Nycticorax nycticorax*)	cloacal swabs	**0 (0)**	**3**	NA	Giacopello et. al. [6]
Netherlands	2010-2011	Grey heron (*Ardea cinerea*)	cloacal swabs/ post-mortem	**2 (18.18)**	**11**	*Escherichia coli* (2)	Veldman et al. [44]
Germany	2011-2014	Black-backed night heron (*Nycticorax nycticorax*)	cloacal swabs	**1**	**NA**	*Escherichia coli*	Schaufler et. al. [39]
			Total	**11 (30)**	**37**		
			Ciconiiformes				
Spain	2001-2002	White stork (*Ciconia ciconia)*	postmortem	**0 (0)**	**3**	*Salmonella* spp.	Millán et al. [21]
	2013-2014	White stork (*Ciconia ciconia*)	cloacal swabs	**3 (33.33)**	**9**	*Escherichia coli* (3)	Alcalá et. al. [29]
	2013	White stork (*Ciconia ciconia*)	cloacal swabs	**114 (95)**	**120**	*Escherichia coli* (106)<br>*Salmonella* spp. (8)	Camacho et. al. [86]
	2018 - 2019	White Ciconia (*Ciconia ciconia*)	cloacal swabs	**22 (22)**	**100**	*Salmonella* Enteriditis (11)<br>*S*. Typhimurium (1)<br>*S*. Typhimurium monophasic variant (mST) (3)<br>*S*. Chester (2)<br>*S*. Infantis (1)<br>*S*. Kentucky (1)<br>*S*. Abony (2)<br>*S*. Pomona (1)	Martin-Maldonado et al. [56]
	2019	White Ciconia (*Ciconia ciconia*)	buffers on the bone surface of fractures	**8**	**4**	*Enterobacter kobei (1)*<br>*Escherichia fergusonii (5)*<br>*Escherichia marmotae (1)*<br>*Shigella flexneri (1)*	Tardón et al. [36]
Italy	2013	White Ciconia (*Ciconia ciconia*)	cloacal swabs	**1 (100)**	**1**	*Escherichia coli*	Giacopello et. al. [6]
Netherlands	2010-2011	Great cormorant (*Phalacrocorax carbo*)	cloacal swabs/ post-mortem	**0 (0)**	**7**	*Escherichia coli*	Veldman et al. [44]
Switzerland	2011-2012	Great cormorant (*Phalacrocorax carbo*)	cloacal swabs	**2 (6.7)**	**30**	*Escherichia coli* (2)	Zurfluh et al. [57]
Austria	2013-2014	Northern bald ibis (*Geronticus eremita*)<br>Grey heron (*Ardea cinerea*)<br>White stork (*Ciconia ciconia*)	cloacal swabs/ faeces/ postmortem	**5 (41.67)**	**12**	*Escherichia coli* (5)	Konicek et. al. [46]
Czech Republic	2013-2014	Grey heron (*Ardea cinerea*)<br>White stork (*Ciconia ciconia*)	cloacal swabs	**17 (73.91)**	**23**	*Escherichia coli* (17)	

Poland	2011-2013	Great cormorant (*Phalacrocorax carbo*)	cloacal swabs	**8 (10.4)**	**77**	*Salmonella enterica (7)*	Krawiec et al. [45]
	2011-2013	Great cormorant (*Phalacrocorax carbo)*	cloacal swabs	**21 (100)**	**21**	*Escherichia coli* (21)	Kuczkowski et. al. [78]
	2011-2014	Great cormorant (*Phalacrocorax carbo*)	NA	**1**	**NA**	*Salmonella enterica* subsp. salamae	Krawiec et al. [58]
			Total	**202 (49.6)**	**407**		
			Gruiformes				
Italy	1991	Black coot (*Fulica atra*)	intestinal content	**1 (10)**	**10**	*Yersinia aldovae*	Iannibelli et al. [81]
Czech Republic	1992-1993	Black coot (*Fulica atra*)	cloacal swabs	**1 (3.33)**	**3**	*S. typhimurium*	Hubálek et al. [67]
Spain	2001-2002	Common moorhen (*Gallinula chloropus)*	postmortem	**0 (0)**	**1**	*Salmonella* spp.	Millán et al. [21]
	2008-2011	Black coot (*Fulica atra*)	cloacal swabs	**0 (0)**	**41**	*Salmonella* spp.	Antilles et al. [77]
	2013-2014	Common crane (*Grus grus*)	cloacal swabs	**0 (0)**	**1**	*Escherichia coli*	Alcalá et. al. [29]
Netherlands	2010-2011	Rail (*Rallidae*)	cloacal swabs/ post-mortem	**0 (0)**	**15**	*Escherichia coli*	Veldman et al. [44]
Poland	2011-2013	Black coot (*Fulica atra*)	cloacal swabs	**0 (0)**	**7**	*Salmonella enterica spp.*	Krawiec et al. [45]
Austria	2013-2014	Cranes (*Antigone canadensis*)	cloacal swabs/ post-mortem	**3 (100)**	**3**	*Escherichia coli* (3)	Konicek et. al. [46]
			Total	**5 (6.2)**	**81**		
			Charadriiformes				
Norway	1980-1981	Puffin (*Fratercula arctica*)	cloacal swabs	**0 (0)**	**76**	*Yersinia* spp.<br>*Salmonella* spp.	Kapperud & Rosef [48]
	1969-2000	Common Murre (*Uria aalge*)	postmortem	**1**	**NA**	*Salmonella enterica* serovar Typhimurium O:4,12	Refsum et al. [17]
	2010	Little auk (*Alle alle*)	cloacal swabs	**NA**	**215**	*Enterobacter cloacae*<br>*E. coli*<br>*Salmonella spp.*	Literak et al. [65]
Spain	2001-2002	Lapwing (*Vanellus vanellus*)<br>Common murre (*Uriaa alge*)<br>Stone curlew (*Burhinus oedicnemus*)	postmortem	**0 (0)**	**4**	*Salmonella* spp.	Millán et al. [21]
Italy	2002-2010	Woodcock (*Scolopax rusticola*)	cloacal swab	**0**	**NA**	*Salmonella enterica*	Botti et al. [42]
Netherlands	2010-2011	Auk (*Alcidae*)<br>Eurasian oystercatcher (*Hematopus ostralegus*)<br>Northern lapwing (*Vanellus vanellus*)<br>Ruff (*Philomachus pugnax*)	cloacal swabs/ postmortem	**10 (15.9)**	**63**	*Escherichia coli* (10)	Veldman et al. [44]

		Sandpiper (*Scolopacidae*)					
Ireland	2013-2021	Eurasian oystercatcher (*Haematopus ostralegus*)	faeces	**NA**	**14**	*Escherichia coli*	Martin et al. [83]
			Total	**11 (3)**	**372**		
			Suliformes				
Netherlands	2010-2011	Northern gannet (*Morus bassanus*)	cloacal swabs/ postmortem	**2 (66.67)**	**3**	*Escherichia coli* (2)	Veldman et al. [44]
			Total	**2 (66.67)**	**3**		
			Procellariiformes				
Spain	2001-2002	Northern fulmar (*Fulmarus glacialis)*	postmortem	**1 (100)**	**1**	*Salmonella* spp.	Millán et al. [21]
Netherlands	2010-2011	Northern Fulmar (*Fulmarus glacialis*)	cloacal swabs/ post-mortem	**0 (0)**	**3**	*Escherichia coli*	Veldman et al. [44]
France (Reunion Island)	2011-2013	Audubon's Shearwater (*Puffinus lherminieri*)<br>Barau's Petrel (*Pterodroma baraui*)	cloacal swabs/ postmortem	**1 (3.45)**	**29**	*Salmonella* spp. serotype 4,[5],12:i:-	Tessier et. al. [59]
			Total				
			Phaethontiiformes				
France (Reunion Island)	2011-2013	White-tailed Tropicbird (*Phaethon lepturus*)	cloacal swabs/ postmortem	**1 (33.3)**	**3**	*Salmonella* spp. serotype 4,[5],12:i:-	Tessier et. al. [59]
			Total	**1 (33.3)**	**3**		
			Podicipediformes				
Netherlands	2010-2011	Great crested grebe (Podiceps cristatus)	cloacal swabs/ postmortem	**0 (0)**	**2**	*Escherichia coli*	Veldman et al. [44]
			Total	**0 (0)**	**2**		
